# Identification and Quantification of Glycans in Whole Cells: Architecture of Microalgal Polysaccharides Described by Solid-State Nuclear Magnetic Resonance

**DOI:** 10.1021/jacs.1c07429

**Published:** 2021-11-04

**Authors:** Alexandre Poulhazan, Malitha C. Dickwella Widanage, Artur Muszyński, Alexandre A. Arnold, Dror E. Warschawski, Parastoo Azadi, Isabelle Marcotte, Tuo Wang

**Affiliations:** † Department of Chemistry, University of Quebec at Montreal, Montreal H2X 2J6, Canada; ‡ Department of Chemistry, Louisiana State University, Baton Rouge, Louisiana 70803, United States; § Complex Carbohydrate Research Center, University of Georgia, Athens, Georgia 30602, United States; ∥ Laboratoire des Biomolécules, LBM, CNRS UMR 7203, Sorbonne Université, École Normale Supérieure, PSL University, 75005 Paris, France

## Abstract

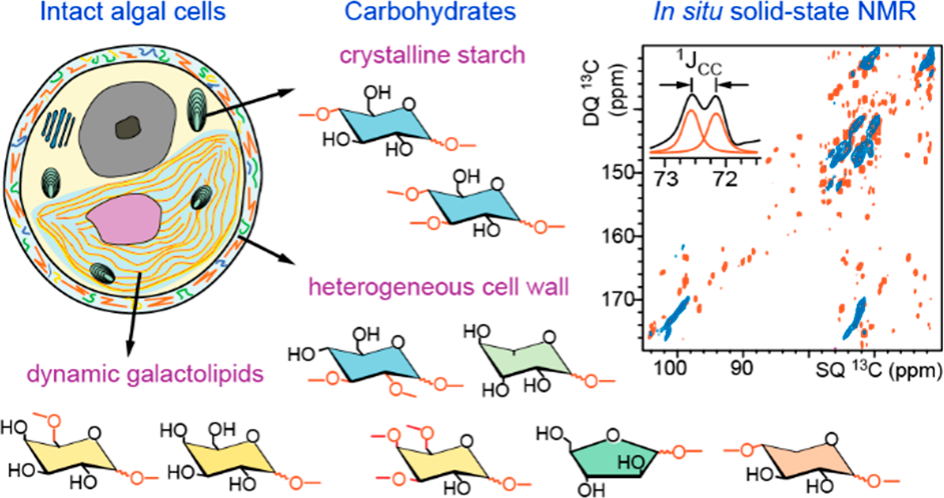

Microalgae are photosynthetic organisms widely distributed in nature and serve as a sustainable source of bioproducts. Their carbohydrate components are also promising candidates for bioenergy production and bioremediation, but the structural characterization of these heterogeneous polymers in cells remains a formidable problem. Here we present a widely applicable protocol for identifying and quantifying the glycan content using magic-angle-spinning (MAS) solid-state NMR (ssNMR) spectroscopy, with validation from glycosyl linkage and composition analysis deduced from mass-spectrometry (MS). Two-dimensional ^13^C–^13^C correlation ssNMR spectra of a uniformly ^13^C-labeled green microalga *Parachlorella beijerinckii* reveal that starch is the most abundant polysaccharide in a naturally cellulose-deficient strain, and this polymer adopts a well-organized and highly rigid structure in the cell. Some xyloses are present in both the mobile and rigid domains of the cell wall, with their chemical shifts partially aligned with the flat-ribbon 2-fold xylan identified in plants. Surprisingly, most other carbohydrates are largely mobile, regardless of their distribution in glycolipids or cell walls. These structural insights correlate with the high digestibility of this cellulose-deficient strain, and the in-cell ssNMR methods will facilitate the investigations of other economically important algae species.

## Introduction

Microalgae are unicellular species widely distributed in all ecosystems on Earth, forming the basis of the aquatic food chain. These photosynthetic organisms capture carbon dioxide and transform it into storage and structural carbohydrates, whose structure and organization differ markedly from those found in terrestrial plants.^1^ With growing economic importance, microalgae have shown attractive applications in the production of biopolymers, the food industry, and environmental applications such as wastewater decontamination.^2−4^ Significant efforts have also been devoted to the development of algae-based third-generation biofuel to circumvent the use of agricultural feedstocks and the competition for land. However, the current algal biofuel still lacks energy- and cost-competitiveness.^5,6^ An in-depth understanding of the structure and assembly of microalgal carbohydrates has the potential of facilitating the optimization of algal systems to produce biopolymers, energy, and food. The identification of the structural characteristics of highly digestible strains and corresponding carbohydrate components could guide the genetic engineering of algal strains to improve cell wall breakdown and enhance the production of fermentable glyco-components or biopolymers of interest. Although some crystalline components (such as starch) have been investigated before, the high-resolution structure determination of other biomolecules in native algal cells, and particularly the highly diverse carbohydrates in the cell wall, remains a formidable problem.^7^

**Figure 1:**
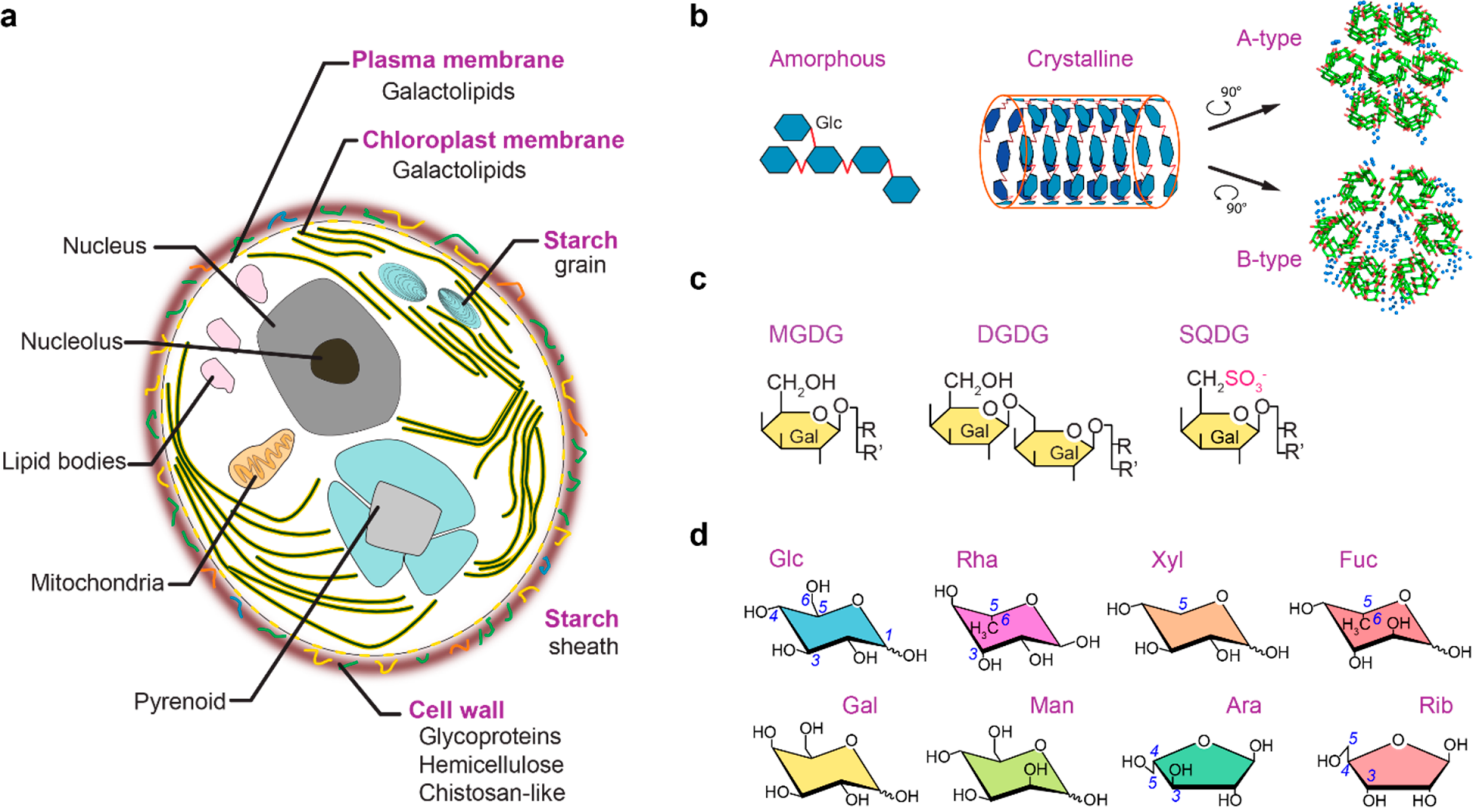
Microalgal carbohydrates found in starch grains, lipids, and cell walls. (a) Main components of microalgal cells. Carbohydrate-rich constituents (purple) include starch in grains, the galactolipids in the chloroplast and cytoplasm membranes, and polysaccharides or glycoproteins in the cell wall. Representative structures of algal carbohydrates are given in panels b–d. (b) Starch can be found in the amorphous or crystalline forms. The crystalline starch fraction is made of 6-fold double-stranded amylose helices, which packing and arrangement lead to two types of starch,^16^ i.e., the A- and B-types as revealed by X-ray crystallography.^17,18^ Amylose polysaccharide helices (green/red for carbon/oxygen) are less tightly packed and more hydrated (blue spheres) in the B-type, as compared to A-type starch. (c) The chloroplast inner membranes and other lipid bilayers contain mono- and digalactosyldiacylglycerol (MGDG, DGDG) and sulfoquinovosyldiacylglycerol (SQDG). (d) Cell walls in *Chlorella* and related algal species are rich in glucose (Glc), rhamnose (Rha), xylose (Xyl), ribose (Rib), galactose (Gal), mannose (Man), arabinose (Ara), and fucose (Fuc). Carbon numbers are given in full for Glc and only at key sites for other residues, with carbons 5/6 being the last carbon of a furanose/pyranose hexose unit.

Carbohydrates in microalgae are mainly present in starch grains, glycolipids, and cell wall constituents (Figure 1a). Up to 60% of the dry weight of a microalgal cell can be made of starch, which is polymerized from α-1,4-glucose and α-1,6-glucose units. Starch can be found in crystalline or amorphous forms, thus dictating the bioavailability of this storage molecule (Figure 1b).^8^ The synthesis and bioaccumulation of starch are regulated by the cell growth stage as well as external stress such as nitrogen deprivation, salinity, and oxidative stress.^9−11^ Genomics approaches have been employed to increase the production of starch in microalgae.^12^ Glycolipids contain oligosaccharides such as mono- and digalactosyldiacylglycerol (MGDG and DGDG) as well as sulfoquinovosyldiacylglycerol (SQDG) (Figure 1c). In the plasma membrane, these lipids are important for cellular recognition and communication, while in the chloroplast membranes, they are essential to photosynthesis. Microalgal cell walls are substantially under-investigated compared to their plant counterparts.^1,13^ Eight monosaccharide types have been reported in the cell walls of the best-studied green microalga *Chlorella*, including glucose (Glc), galactose (Gal), rhamnose (Rha), mannose (Man), xylose (Xyl), arabinose (Ara), fucose (Fuc), and ribose (Rib), each of which could exist in either the furanose (*f*) or pyranose (*p*) forms, with variability of linkage patterns and methylated sites^14,15^ (Figure 1d), thus leading to a tremendous diversity in terms of overall cell wall composition and architecture.

The characterization of microalgal and plant glycans has typically been conducted using radiolabeling,^19^ gas chromatography and mass spectrometry,^20−24^ as well as solution^25^ or high-resolution magic-angle spinning (HRMAS)^26,27^ NMR methods. These approaches provide valuable information on glycan composition and connectivity but often require hydrolysis or solubilization procedures that compromise the physical and structural complexity of native biomolecules. Cellular samples can be assessed using in situ solid-state nuclear magnetic resonance (ssNMR), an emerging nondestructive method with atomic to nanoscale resolution complementing X-ray diffraction and electron microscopy techniques.^28^ SsNMR has enabled high-resolution characterization of bacterial cells,^29−34^ plant tissues,^35,36^ fungal mycelia and cells,^37−41^ mammalian cells,^42^ viral particles,^43,44^ and recently algal cultures^45−47^ under MAS conditions.

Here we established an MAS ssNMR protocol, aided by gas chromatography–mass spectrometry (GC-MS), to quantify the carbohydrate components and determine the glycans’ architecture using ^13^C-labeled microalgal cells. This approach is based on 2D MAS ssNMR spectra and has never been employed for algal cell samples. Although complete quantification of the carbohydrate composition has been recently conducted in plant polysaccharides,^48^ it was primarily based on 1D methods with limited resolution. 2D MAS ssNMR experiments are typically conducted to examine polymer interactions and distinguish molecules in mobile or rigid phases, but have rarely been used for quantification.^49,50^ Therefore, this study presents advances in both the methodology and cell types being investigated.

We demonstrated the approach on a strain of green microalgae (*CK-5*), which was first identified as *Chlorella vulgaris* based on its morphology,^51^ but later renamed *Parachlorella beijerinckii* following genomic sequencing.^52^*CK-5* naturally shows a negligible cellulose content. This strain has also unique capabilities of producing high-value fermentable glycans and more importantly secreting polysaccharides attached to the cell surface,^53,54^ resulting in a soft and digestible cell wall that facilitates oil extraction and increases bioavailability.^55,56^ This strain can also effectively eliminate heavy metals,^49−51^ which is of great interest for phytoremediation processes.

The carbohydrate composition and glycan structures of *Parachlorella* and this important *CK-5* strain has yet to be determined. So far, information can only be deduced from *Chlorella*, another genus within the same *Chlorellaceae* family. The *Chlorella* genus is also debated, even if this group is described as pyrenoid-containing alga-clade, and is one of the most-studied phototrophic eukaryotes, with more than 100 strains described, compassing 37 taxonomically acknowledged species^57^ found in freshwater, marine, and soil habitats.^58,59^ In general, the most abundant monosaccharides identified in Chlorella and *Parachlorella* cell walls are glucose, galactose, and rhamnose,^13,60^ which are glycan units commonly associated with cell surface interactions in algae and in bacteria.^61,62^ Most publications agree that *Chlorella* microalgae generally contain cellulose (rich in Iα allomorph) and alkali-soluble hemicellulose,^63−65^ but other assemblies have also been reported, such as acidic polysaccharides containing arabinogalactan, glycoproteins, phosphorylated β-galactan, as well as chitin-like^66^ and other glycan-rich cell walls.^64,67−71^

With surprisingly high resolution that is even sufficient to resolve ^13^C–^13^C *J*-couplings in-cell, we assigned the signals of 7 monosaccharide types and resolved 58 of their subforms (with 37 assigned, 17 tentatively assigned, and 4 unknown spin systems). We further examined the composition, dynamical characteristics, and spatial organization of these assigned glycans. Starch was identified as the major rigid and predominantly abundant molecule in *Parachlorella CK-5*. The starch grain of this strain appears to exist in a well-organized form as revealed by the strong inter-residue and intermolecular interactions between different glucose conformers and between the major starch domains and associated segments. Some xyloses were distributed in both rigid and mobile domains, and their chemical shifts suggest a conformation similar to the flat-ribbon 2-fold xylan (two sugars per helical turn) identified in plants.^36,72^ Most other carbohydrate components were found to be dynamic, not only in glycolipids but also in the cell walls. As *Chlorella* and *Chlorella*-like algae are important model microorganisms carrying a high amount of proteins and carbohydrates, an in-depth understanding of their glycan architecture will shed light on the structural mechanisms of their resistance to microbial and chemical degradation,^73^ which are important parameters to consider in biopolymers production. The approaches established here could be applied to many algal strains with a variable content of cellulose, where the cell wall network hinders the efficient access and extraction of molecules for the production of biofuels, biopolymers, bioplastics, antioxidants, and decontaminants.^74−76^

## Experimental Section

### Microalgal Sample

Freeze-dried uniformly ^13^C-labeled *P. beijerinckii CK-5* (SAG 2046) microalgae were prepared by Iso-Life (Wageningen, The Netherlands) using confidential autotrophic growth conditions. The algal strain *Parachlorella beijerinckii* (or *beyerinckii*) *CK-5* was developed for industrial purposes and associated with the *Chlorella* genus, and named Chikugo (*CK*) after the district of the Fukuoka prefecture in Japan. The strain has been later identified and classified based on the sequences of its 18S ribosomal ribonucleic acid gene (AY323841^52^), internal transcribed spacer (ITS FM205845^77^ and ITS-2 AY323476^52^), and other morphological characteristics.^52^ About 30 mg of dried cells were rehydrated with 70 μL of nanopure water, and the whole-cell material was packed into a 3.2 mm MAS rotor for ssNMR characterization. To evaluate the effect of freeze-drying on microalgal samples, we compared fresh and hydrated *C. reinhardtii* cells to a lyophilized and rehydrated sample (Figure S1). There is no significant perturbation of the glycan composition, and the dynamics of most microalgal molecules was efficiently retained. This is consistent with the recent observation that freeze-drying and rehydration have negligible effect on the carbohydrate structure and dynamics in the model plant *Arabidopsis*,^78^ or on *Escherichia coli* bacterial membranes.^79^

### Glycosyl Composition and Linkage Analysis by Mass Spectrometry

Glycosyl composition analysis of neutral sugars was achieved after an acetone extraction on dried cells, removing most pigments in the sample. The dried sample was then dispersed in 0.5 mL of 2 M trifluoroacetic acid (TFA) in a sealed reaction tube. After 20 min of sonication in an ultrasound water bath at room temperature, hydrolysis was performed at 121 °C for 2 h, followed by overnight reduction with NaBD_4_, and 1 h acetylation with acetic anhydride and pyridine (1:1, v/v) at 80 °C.

GC-MS analyses were performed with an HP-5890 GC interfaced to a mass-selective detector 5970 MSD using a SupelcoSP2330 capillary column (30 × 0.25 mm ID, Supelco) with the following temperature program: 60 °C for 1 min, then ramp to 170 °C at 27.5 °C/min, and to 235 °C at 4 °C/min, with 2 min hold, and finally to 240 °C at 3 °C/min with 12 min hold. Inositol was used as an internal standard. Glycosyl constituents were assigned, based on the GC-MS retention time of the sugar standards derivatives, and on electron ionization MS (EI-MS) fragments of ^13^C-labeled alditol acetate derivatives. Results of this quantitative GC-MS glycan composition are shown in Table S1, and the interpretation of the EI-MS fragmentation is given in Figure S2.

The glycosyl linkages of uniformly ^13^C-labeled polysaccharides were obtained by GC-MS of partially methylated alditol acetates (PMAA)^80^ after 2 h of hydrolysis with 2 M (v/v) TFA at 121 °C, overnight reduction with NaBD_4_ and acetylation with acetic anhydride and pyridine. Inositol was used as an internal standard. Detected glycan linkages are given in Table S2. The examples of GC-EI-MS analysis of ^13^C-labeled PMAAs molecules and the interpretations of EI-MS fragmentation have been recently demonstrated on fungal cell-wall carbohydrates.^37^

### Solid-State NMR Experiments

All experiments were conducted at the National High Magnetic Field Laboratory (Tallahassee, FL, USA) on an 800 MHz (18.8 T) Bruker Avance III HD NMR spectrometer using a high-efficiency 3.2 mm HCN probe under 13.5 kHz MAS at 27 °C. ^13^C chemical shifts were externally referenced to adamantane’s CH_2_ signal set to 38.48 ppm.^81^ The typical radiofrequency (RF) field strength was 83 kHz for ^1^H decoupling, using the two-pulse phase-modulated (TPPM) scheme, with 5.7 μs for each pulse. The RF field strength was 83 kHz for ^1^H hard pulses, 62.5 kHz for ^13^C hard pulses, and 50 kHz for both ^1^H and ^13^C cross-polarization (CP) spin lock. The key acquisition parameters of all 1D and 2D experiments are summarized in Table S3. Furthermore, all the assigned peaks will be deposited to the Complex Carbohydrate Magnetic Resonance Database (CCMRD),^82^ and the Bruker Topspin data set is freely available upon request.

To probe polysaccharides with different dynamics, four 1D ^13^C spectra were acquired using different schemes for creating initial magnetization. ^13^C direct polarization (DP) spectra were acquired using either a 30 s or a 2 s recycle delay for quantitative detection of all molecules or selective highlight of mobile molecules, respectively. For rigid molecules, CP was used with a 1 ms contact time. Refocused Insensitive Nuclei Enhanced by Polarization Transfer (INEPT) experiments with two delays of 1.72 ms followed by two delays of 1.15 ms were employed to probe mobile species.

To assign glycan signals, 2D ^13^C DP refocused J-INADEQUATE^83,84^ spectrum was collected using a recycle delay of 2 s. ^13^C CP refocused J-INADEQUATE was also employed to detect rigid molecules, using the CP parameters described above for 1D analogs. INADEQUATE acquisition and processing parameters have been described before,^84,85^ with each delay during the polarization transfer set to 2.4 ms. To directly compare INADEQUATE—a double quantum (DQ) correlation experiment—with the single quantum (SQ) spectra described below, the INADEQUATE spectrum was sheared^86^ using the “ptilt1” command available on Bruker TopSpin software. The resonance assignments of 37 carbohydrates subforms are displayed in Table S4, and compared with literature values in Table S5. The tentative assignments of 17 carbohydrates subforms as well as 4 unassigned forms are listed separately in Table S6 to avoid confusion.

To probe internuclear correlations with a progressively increasing distance range, three types of 2D SQ ^13^C–^13^C correlation experiments were conducted, including RF-Driven Recoupling (RFDR) with a short recoupling time of 1.5 ms that mainly selects one-bond cross peaks, 53 ms COmbined R2^v^_n_-Driven recoupling (CORD)^87,88^ that reports intraresidue correlations, and 14 ms Proton Assisted Recoupling (PAR)^89^ that permits the identification of long-range inter-residue and intermolecular cross peaks. The ^13^C and ^1^H RF field strengths of the PAR mixing were set to 53 and 50 kHz, respectively.

## Results and Discussion

### Screening the Dynamical Distribution of Biomolecules in Intact Algal Cells

**Figure 2:**
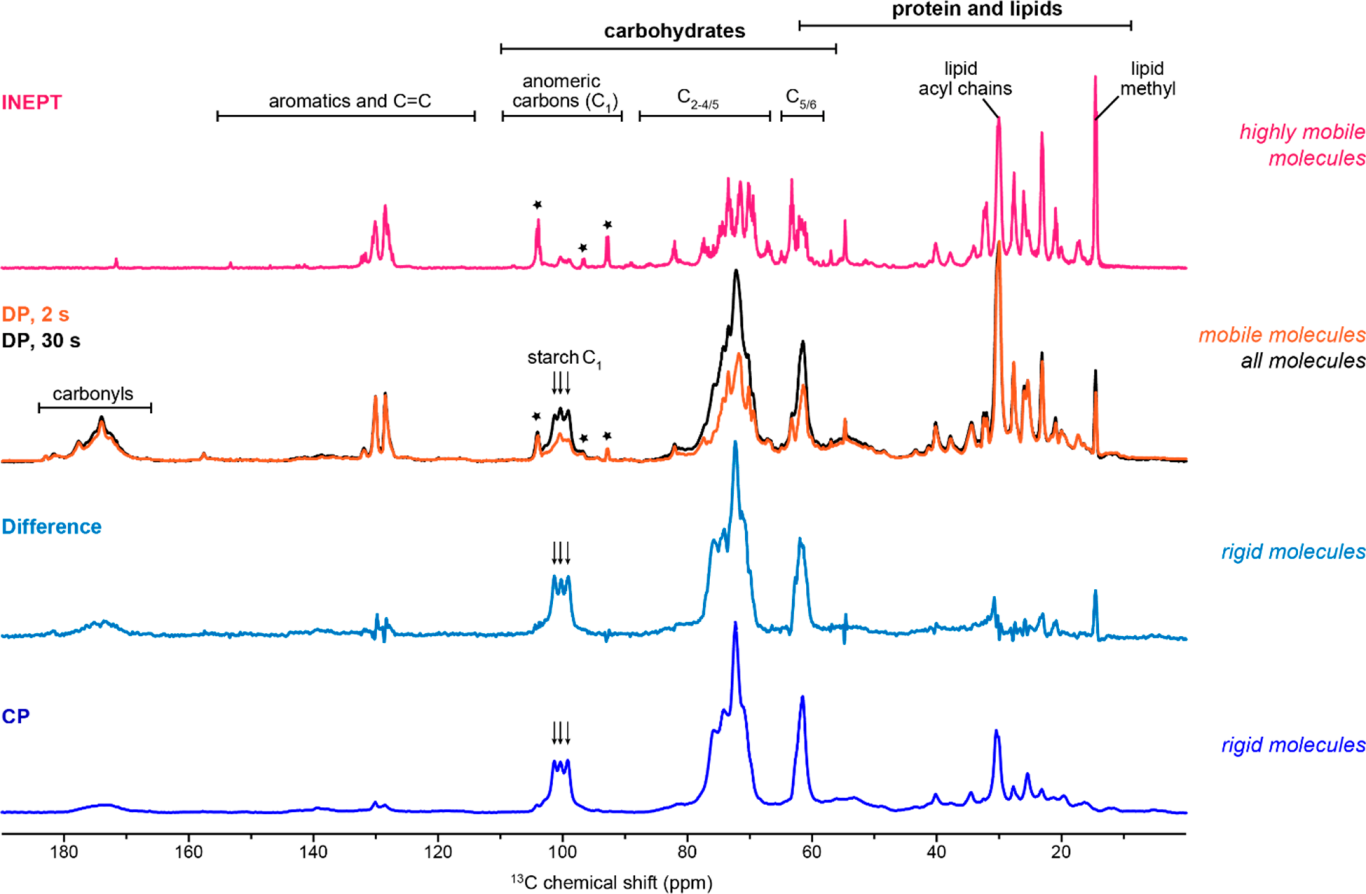
Dynamic heterogeneity of microalgal molecules revealed by 1D ^13^C ssNMR spectra. The *J*-coupling-based refocused INEPT spectrum (magenta) only shows signals of highly mobile molecules. ^13^C DP spectra with a long recycle delay (30 s; black) enable quantitative detection while a short recycle delay (2 s, orange) detects mobile components. Arrows and stars are respectively used to highlight the carbon 1 sites of rigid starch and C1 of mobile carbohydrates. The difference spectrum (light blue) of the two DP parental spectra is highly similar to the CP spectrum (blue), selecting rigid molecules. All spectra were collected using intact *P. beijerinckii* cells on an 800 MHz NMR spectrometer, with 13.5 kHz MAS frequency and 83 kHz TPPM ^1^H decoupling.

The sample analyzed by MAS ssNMR was uniformly ^13^C-labeled and hydrated *P. beijerinckii* cells, free of chemical treatment and extraction. The distribution of microalgal biomolecules in different dynamical regimes was rapidly assessed using a series of 1D ^13^C spectra (6–30 min each). The most mobile molecules or structural motifs were selected via a *J*-coupling-based INEPT experiment (Figure 2). The concerted motions of wobbling and uniaxial rotation in lipids allowed their signals to be detected, such as the acyl chain CH_2_ peak at 30.1 ppm, methyl signal at 14.6 ppm, and the olefin carbons in unsaturated lipids (128 to 130 ppm). Carbohydrate signals were also observed, such as C_1_ of xylose and galactose (ca. 104 ppm), indicative of their presence in a largely solvated state. A ^13^C DP spectrum using a sufficiently long (30 s) recycle delay allowed the quantitative detection of all carbon sites in the algal cells. Signals from rigid components, for example, the three C_1_ peaks of glucose in starch (99, 100, and 101 ppm assigned as S1^a^, S1^b^, and S1^c^, respectively),^90^ are sequentially increased from the INEPT to the two DP spectra collected with 2 and 30 s recycle delays. This is not surprising since the use of a short recycling time suppresses the intensities of rigid molecules due to their slow ^13^C-T_1_ longitudinal relaxation, while a sufficiently long recycle delay allows re-equilibration of the magnetization and quantitative detection of all carbons. Although both INEPT and 2 s DP preferentially highlight mobile molecules, the proton-to-carbon polarization transfer pathway of INEPT made it fail to detect nonprotonated carbons, such as the protein/lipid carbonyl groups, which showed to be mobile from their presence in the 2 s DP spectrum.

Rigid components can be selectively observed using either a difference spectrum generated by subtracting the 2 s DP spectrum from the 30 s DP spectrum, or through a proton-to-carbon CP experiment. The definition of rigid is different in these two methods, with the former applying a relaxation (^13^C-T_1_) filter and the latter based on dipolar-coupling mediation. This explains the discrepancy in the protein and lipid regions when comparing the two spectra. A consistent observation is the dominance of starch peaks, thus establishing this biopolymer as the major rigid carbohydrate in *P. beijerinckii CK5*. Therefore, the 1D approach allowed us to either quantitatively detect all molecules in a cell or select components according to their dynamical characteristics.^45,46^ Such versatility enables the design of 2D ssNMR experiments and analysis methods to assign and quantify the structurally complex glycans.

*P. beijerinckii* is particularly rich in carbohydrates, as shown by the comparison with two cellulose-producing species (such as *Nannochloropsis oculata* and *Pavlova lutheri*) and the glycoprotein-rich *C. reinhardtii* (Figure S3a,b). A partial similarity in carbohydrate structures can also be noticed between *P. beijerinckii* and *C. reinhardtii* (Figure S3c). It is of significant interest to investigate the similarities and differences in the compositions and structures of carbohydrates found in cellulose-rich and cellulose-free algal strains.

### Resonance Assignment of Glycans

**Figure 3:**
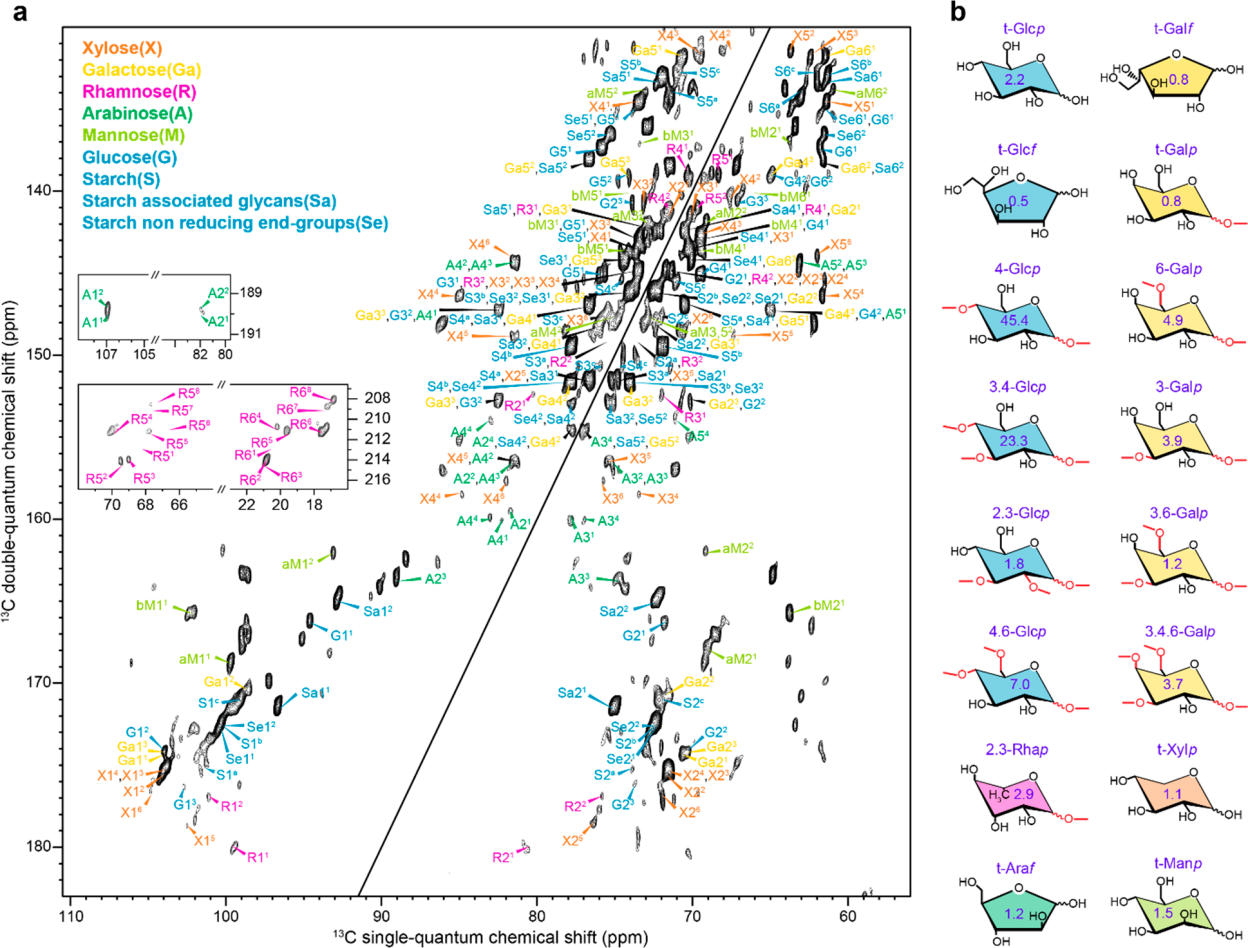
Complexity of carbohydrates structures resolved with 2D correlation spectra. (a) ^13^C refocused DP-based *J*-INADEQUATE spectrum of rehydrated uniformly ^13^C-labeled *P. beijerinckii* hydrated cells with a 2 s recycle delay selecting the mobile moieties of polysaccharides. The spectrum was acquired on an 800 MHz NMR spectrometer under 13.5 kHz MAS using 83 kHz TPPM ^1^H decoupling. Each echo delay was set to 2.4 ms. Abbreviations are used for glycan resonance assignments and color-coding is primarily based on the symbol nomenclature for glycans (SNFG).^91^ The XZ^Y^ notation is used where X is the type of sugar, Y is the spin system number (for example, Ga^1^, Ga^2^, and Ga^3^ for 3 galactose units), and Z is the carbon number (for example, Ga6^1^ is the carbon 6 of galactose 1 unit). Insets show the unique signals of arabinose carbon 1 and rhamnose carbon 6. (b) Representative structures of carbohydrate units with connectivity identified by mass spectrometry. The number in purple indicated for each unit corresponds to the relative EI detector response (percentage) obtained by GC-MS, which is a semiquantitative reporter as provided in Table S2.

To fully assign the glycan signals, 2D ^13^C–^13^C correlation spectra were acquired using a high-field NMR spectrometer (800 MHz). We preferred the refocused *J*-INADEQUATE sequence since it further improves spectral dispersion by the introduction of DQ dimension and the absence of an often-crowded diagonal. The refocused DP-*J*-INADEQUATE spectrum of *P. beijerinckii* is shown in Figure 3a with all the spin systems labeled and compared to the glycan connectivities identified by glycosyl composition and linkage analysis (Figure 3b). The assignment of more than 35 spin systems (Tables S4 and S5) was facilitated by comparison with the glycan chemical shifts indexed in the Complex Carbohydrate Magnetic Resonance Database.^82^ Arabinose signals were readily assigned due to the C_1_ chemical shift close to 110 ppm (A1^1^, A1^2^, A1^3^, and A1^4^), as well as rhamnose which C_5_ signal appears at ca. 20 ppm (R5^*x*^, with *x* from 1 to 8) (Figure 3a, insets). Galactose, on the other hand, was identified by comparison with resonance patterns (C_1_ to C_5_ ring in galactofuranose or C_6_ in galactopyranose) determined on simpler model compounds. Glucose in starch was easily assigned as it is predominant in this sample, and by comparing its signals to those reported in other cells.^90^ Since 1D results defined starch as the major rigid molecule in this cell (Figure 2), its assignment was also confirmed with a CP-based refocused *J*-INADEQUATE spectrum (Figure S4).

The assignment of the remaining glycans proved to be much more arduous due to the structural complexity and overlapping of polysaccharides. Nevertheless, we could identify the six principal monosaccharide units detected by MS approaches, i.e., arabinose, galactose, glucose, mannose, rhamnose and xylose, in 4, 3, 11, 3, 8, and 6 environments, respectively (Figure 3 and Table S4). Representative spin correlations pathways of these molecules are shown in Figure S5. These glycans are mainly associated with starch metabolism but are also found in galactolipids located in the chloroplast membranes, and glycoproteins or polysaccharides from the cell wall. Dovetailing with the 1D results, both refocused DP and CP-INADEQUATE spectra reveal three sets of chemical shifts for the anomeric carbon of starch from *P. beijerinckii* cells (Figures 3 and S4), consistent with A-type starch (Figure 1b), as observed in other algae such as *C. reinhardtii*.^90^ The high spectral resolution in this work allows the complete description of all the spin systems in A-type starch in cells.

Although beyond the scope of this work, amino acids in whole microalgae were also detected using the refocused DP-INADEQUATE pulse sequence. They appear to be very mobile as they were almost undetectable with the CP-INADEQUATE experiment (Figure S6). This suggests that individual membrane proteins and proteins involved in the cell-wall network are either dynamic or of low abundance.

### Quantification of Glycans in Whole Cells

To quantify glycans in whole *P. beijerinckii* cells, we relied on the refocused INADEQUATE experiment (Figure 3) due to its exceptional resolution, although it comes with some limitations. Notably, variations in intensity can be expected for carbons involved in different homonuclear *J*-couplings as this experiment exploits ^13^C–^13^C *J*-couplings. Transverse (T_2_) relaxation during the refocused INADEQUATE blocks can vary among different carbon sites^84,92,93^ and affect the intensity of the ^13^C peaks used for quantification. Since T_2_ can be a function of molecular motions, systems with heterogeneous dynamics require careful analysis. Nevertheless, this quantification approach can be applied to quantify molecules in samples with homogeneous dynamics and ^13^C-T_1_ relaxation, providing that the few molecules with different dynamics are calibrated using 1D experiments, which is assumed to be the case with whole *P. beijerinckii* cells. Despite these challenges, glycan quantification can be performed by using a single peak in a given system of correlated peaks (which corresponds to a single glycan) or by averaging the intensities of all correlated peaks. We favored the latter solution to maximize precision and compensate for potential excitation inhomogeneity.

Although ssNMR quantification of carbohydrates has been reported in plant materials using the deconvolution of 1D quantitative ^13^C spectra,^48^ the use of 2D methods, as reported here, is a first to our knowledge. Multidimensional experiments allow the assignment of previously unresolved glycans resonances on 1D spectra, but quantification with 2D spectra remains challenging due to the low chemical shift dispersion of polysaccharides signals. Moreover, accurate quantification can be hindered by the coexistence of both pentoses and hexoses that contribute differently to the spectral intensity because of their different carbon numbers.

**Figure 4:**
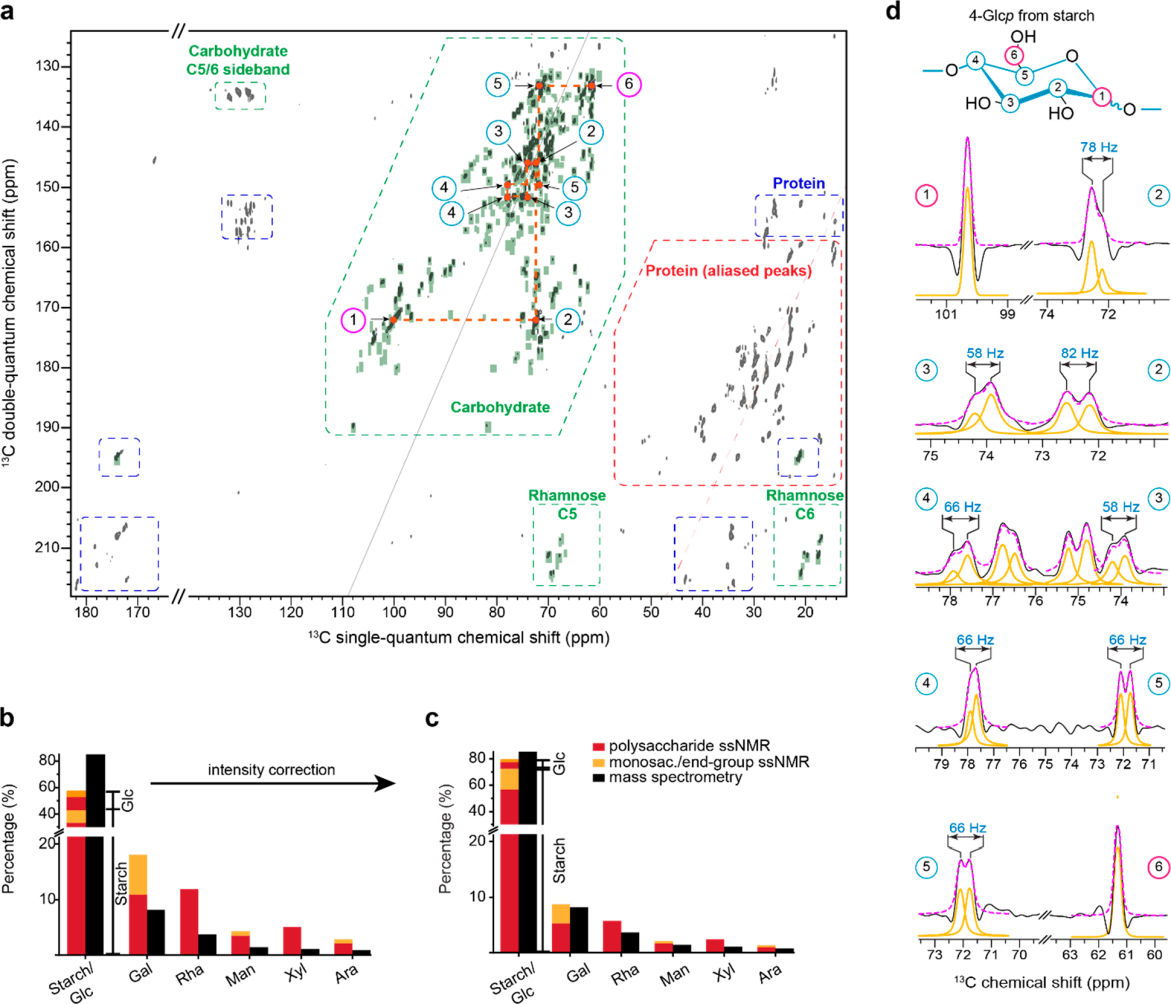
Carbohydrate composition estimated from ssNMR and MS analyses of *P. beijerinckii* rehydrated cells. (a) Integration regions (green rectangles) of the 2D refocused DP-INADEQUATE spectrum used for glycan quantification (see Figure 3 for key experimental details and assignment). (b) Comparison of rehydrated *P. beijerinckii CK-5* glycan composition derived from the 2D spectrum in panel (a) showing significant discrepancy with MS results. Data are summarized in Table S7. The difference is caused by the suppression of starch signals. (c) Good correlation between MS results and the recalibrated ssNMR quantification after correcting for the starch content. A correction factor of 3.5 obtained using 1D spectra (Figure S8) was applied on the starch fraction. (d) Cross sections extracted from the 2D refocused DP-INADEQUATE spectrum showing splittings for starch and most glycans. This observation is associated with ^1^*J*_*CC*_ couplings. The starch slices shown here were deconvoluted (blue), with a good match between the simulated (magenta) and measured (black) spectra. Carbon 1 and terminal 5/6 each showed a single peak while other central carbons showed pairs of peaks.

We first considered simple cases that showed no spectral overlap and integrated all the peaks in a system of correlated peaks (Figure 4a). In the 2D refocused INADEQUATE spectrum, pairs of peaks appear horizontally at the DQ frequency corresponding to the sum of the chemical shifts of the correlated spins. A five-membered ring, for example, will yield the following four pairs of peaks at the various δ_C*n*_ chemical shifts: (δ_C1_, δ_C2_), (δ_C2_, δ_C3_), (δ_C3_, δ_C4_), and (δ_C4_, δ_C5_). The carbons on the extremities of the glycan (C1 and C5) therefore contribute less to the overall intensity than the remaining carbons. This was rectified by normalizing the sum of integrals by the number of peaks in this system, which also corrected for the different contributions of hexoses and pentoses. The relative abundance of a specific glycan (RA^*glycY*^) was therefore calculated as follows:
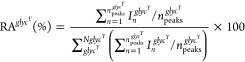
1with  denoting the number of peaks assigned to a specific glycan *glyc*^*Y*^ (ex: 6 peaks with no overlap for Gal^1^) and *Nglyc*^*Y*^ representing the total number of different glycans Y (Ara^1^ + Ara^2^ + ... + Gal^1^ + ... + Glc^1^ + ... + Xyl^1^ + ...). For numerical results, see Table S7, Method 1.

Without spectral overlap, the carbon quantification was easily performed by integrating the corresponding correlation peak volumes. This strategy also enabled the quantification of specific glycans, even distinguishing various glycans of the same nature (ex: Glc^1^ from Glc^2^), as shown in Table S8 where nonoverlapping peaks appear in black. As whole cells comprise multiple complex glycans, there was a significant peak overlap on the spectrum that required another approach. Two types of overlapping peaks could be distinguished: those from glycans of the same nature (Gal^1^ and Gal^2^ for example) and those from different types of glycans (Gal^1^ and Glc^1^ for example). Overlapping peaks are shown in Table S8, highlighted in orange or red if they come from the same type or different types of glycans, respectively.

With overlapping peaks, the precise relative contribution of each glycan to the peak volume cannot be determined, and overlapping peaks originating from different glycans cannot be easily exploited for quantification purposes. However, in the case of overlapping peaks of the same glycan, the total volume can provide the relative abundance of this sugar with respect to other types of glycans. To do so, eq 1 can be used with integrals of each resonance divided by the number of carbons it contains, allowing the consideration of a large number of assigned peaks. Indeed, even if the relative contribution to the integral of each of the overlapping carbons is unclear, the number of carbon resonances and spin system number from the same glycans that are overlapping is known. This leads to the following equation:
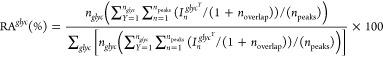
2where *glyc* is the glycan type (Ara, Rha, Man, ...),  is the number of spin systems assigned for a specific glycan type *glyc* (ex: 3 galactose spin systems for Gal^1^, Gal^2^ and Gal^3^), *n*_peaks_ represents the number of unique peaks assigned to a specific glycan type *glyc*^*Y*^ (for example, 17 carbons assigned in galactose that do not overlap with different glycan type), *n*_overlap_ indicates the total number of carbons that overlap for a given integral  (for example, *n*_overlap_ is 0 for galactose), and  is the sum over the different glycan types (Ara; Gal; ...; Xyl).^94,95^ Considering the number of accounted carbon and spin system numbers is important for preventing underestimation of glycan types with many different spin systems but with low integral per spin system (see Table S7, Method 2). The overall flowcharts for these two methods used for glycan quantification are summarized in Figure S7. It should be noted that most previous ssNMR quantification methods rely on spectral deconvolution,^48,96^ and the current method to obtain averaged intensity has rarely been employed.

As demonstrated with the 1D spectra, the quantification of ^13^C signals in whole-cell glycans requires a long recycle delay of 30 s, which results in multiple days to record each 2D spectrum. Therefore, we opted for a recycle delay of 2 s, at the expense of selectively reducing the signal of rigid moieties. As expected, glucose signals in crystalline starch were affected by the short recycle delay (see Figure 2). Starch resonances obtained with the 1D spectra with short (2 s) and long (30 s) recycling delays were therefore used to obtain a correction factor of 3.5 (Figure S8) that was then applied to their intensities in the 2D spectrum. After this recalibration, glycans in *P. beijerinckii CK-5* whole cells could thus be quantified with a recycle delay of 2 s and compared to the more accurate MS quantification, as outlined in Figure 4b,c. Overall, calibration of the ssNMR data provided a better match with the MS results, as compared to uncalibrated intensities. Taking the galactose composition as an example, a good agreement is found between the MS (8.2%) and calibrated ssNMR data (8.5%), which is a significant improvement over the uncalibrated method (17.8%) that corresponds to a 117% relative error. For the least abundant arabinose residues, a proportion of 0.8% was obtained by MS analysis, 1.3% with the calibrated ssNMR method, and 2.8% with the uncalibrated method, corresponding to a 250% relative error (Table S7).

The quantification of glycans performed here on *P. beijerinckii* cells by ssNMR appears as a reliable approach providing the application of appropriate controls. 1D spectra can indicate whether intensity calibration is needed for specific resonances to compensate for the polarization transfer inhomogeneity in the refocused INADEQUATE experiment. A crystalline polysaccharide such as starch will likely not be present in many types of cells and, in a more structurally and dynamically homogeneous sample, correction factors might not be required. Nevertheless, cross validation with MS is advisable. An interesting alternative might be the use of relaxation agents, which could reduce and homogenize longitudinal relaxation throughout the sample.^97^

### Observation of ^1^*J*_*CC*_ Couplings in the Cell

The spectral resolution allowed observing one-bond scalar couplings (^1^*J*_*CC*_) for some carbon sites.^98−100^ Such lineshapes have been observed in proteins for a long time, and the effects of rotational-resonance and coherent cross-correlation under slow MAS frequencies have been discussed in detail.^84,101,102^ To our knowledge, these scalar couplings, which helped carbon assignment, have not been reported in any whole-cell systems, although similar splittings were observed for the most dynamic arabinose units in an *Arabidopsis* cell wall sample.^103^*Parachlorella* might benefit from narrower ^13^C line widths and better-resolved *J*-couplings compared to more rigid systems. Most glycan C_2_-to-C_4/5_ correlation peaks exhibit *J*-splittings with values between 50 and 80 Hz (Table S5, Figure 4d and S9), which are slightly higher than ^1^*J*_*CC*_ couplings measured for oligosaccharides in solution NMR.^104−106^ These larger ^1^*J*_*CC*_ values determined in solids might result from the distribution in dihedral angles in such an inhomogeneous system and a combined effect from several *J*-couplings. Heteronuclear ^1^H–^13^C *J*-couplings are used in solution NMR for the structural determination of glycans. Emerging work is also being carried out to understand the relationship between the homonuclear ^13^C–^13^C *J*-couplings and the structure of glycans in solution^104,105^ and solids.^107^ These couplings helped to alleviate the assignment ambiguities in our sample. Peak splitting is not observed for the terminal sites (such as C_1_ and C_6_ in glucose) that only have a single neighboring carbon but observed for all remaining sites (such as C_2_–C_5_ in glucose) that have two neighboring carbons. This unexpected pattern of splitting is not yet understood and requires further assessment.

### Spatial Proximity of Rigid Carbohydrates

**Figure 5:**
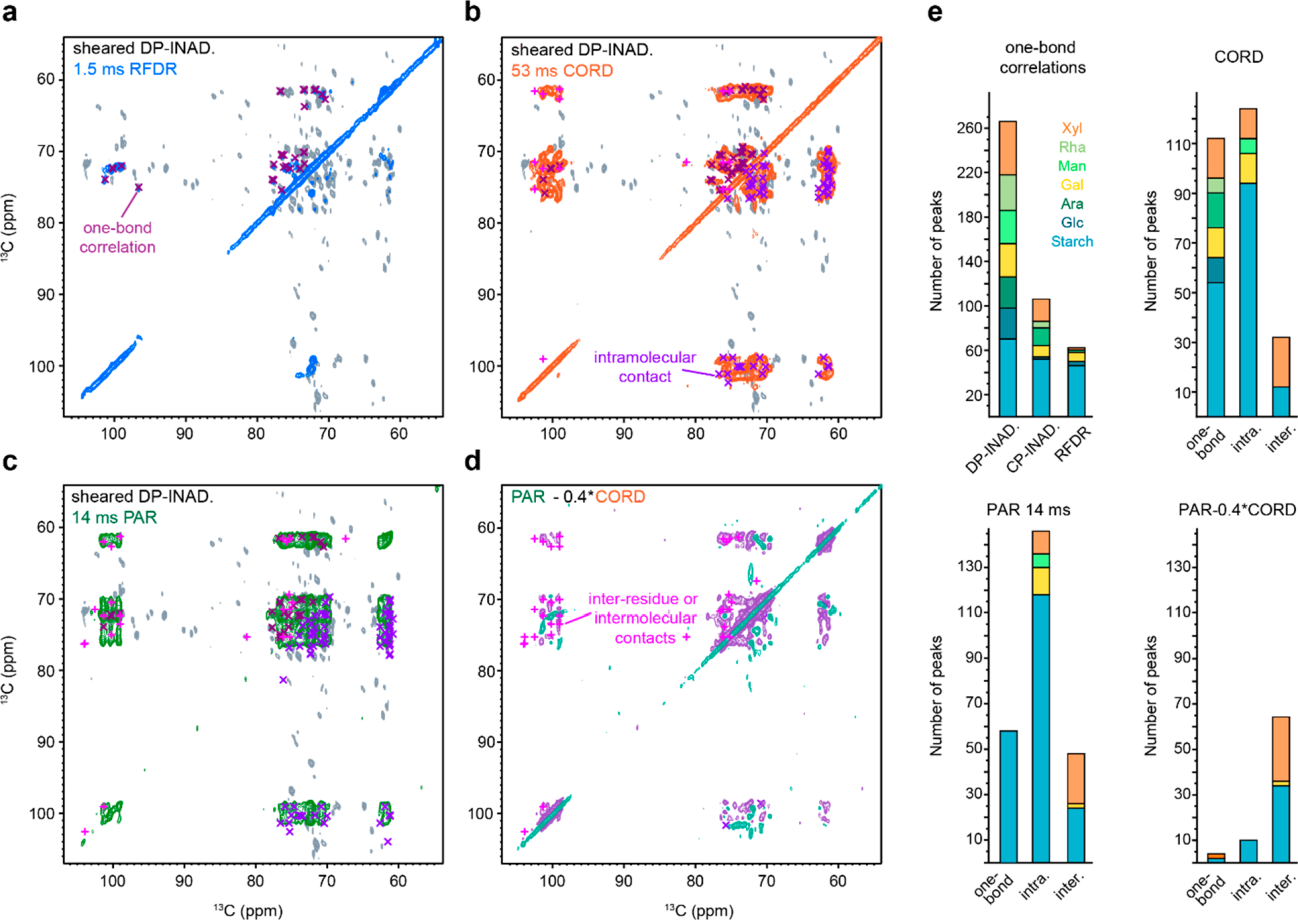
2D ^13^C–^13^C correlation spectra for identifying intra- and inter-residue contacts in rehydrated *P. beijerinckii* cells. Sheared DP-INADEQUATE spectrum (gray) was overlapped with different CP-based spectra (RFDR, CORD, and PAR) that detect rigid molecules. (a) RFDR experiment with a short mixing time of 1.5 ms for selective detection of one-bond correlations. (b) 53 ms CORD detecting most of the intramolecular cross peaks. (c) 14 ms PAR showing long-range intra- and intermolecular cross peaks. (d) Subtraction of the CORD spectrum from the PAR spectrum resolving intermolecular contacts between starch and cell wall components. A scaling factor of 0.4 was applied to the CORD spectrum to cleanly remove all intramolecular cross peaks. All the CP-based spectra are dominated by starch, together with some of the galactose, xylose, and mannose units. One-bond correlations (dark purple), multibond intramolecular cross peaks (light purple), and inter-residue or intermolecular contacts (magenta) are marked and color-coded. (e) Numbers of peaks observed in different 2D spectra. The signals involved in one-bond correlations and their sugar types are shown for refocused DP- and CP-INADEQUATE, and RFDR spectra (top left). One-bond correlation peaks, multibond intramolecular cross peaks, and inter-residue or intermolecular contacts are shown for CORD, PAR, and their difference spectrum. All spectra were acquired with an 800 MHz NMR spectrometer under 13.5 kHz MAS frequency using 83 kHz TPPM ^1^H decoupling.

The spatial association of glycans was examined using three different 2D ^13^C–^13^C correlation experiments probing progressively shorter internuclear distances (Figure 5). With a focus on rigid molecules via CP, one-bond correlations were selectively detected using a 1.5 ms RFDR experiment (Figure 5a) while all intramolecular correlations (including one-bond and multibond cross peaks) were observed using a 53 ms CORD spectrum (Figure 5b). The 2D PAR experiment was designed to determine long-range (∼ca. 5 to 10 Å) and intermolecular or inter-residue contacts,^89,108,109^ and, in the case of *Parachlorella*, showed mostly starch in addition to a few xylan correlations (Figure 5c and Table S9). Indeed, the PAR experiment has been extensively used in the structural determination of proteins and amyloid fibrils^89,108−112^ and was recently applied to cell wall materials.^37^ In addition, the difference of the PAR and CORD spectra (generated by spectral subtraction) allowed the unambiguous observation of intermolecular cross peaks (Figure 5d),^113^ and even detect contacts that were not visible in the PAR spectrum due to spectral crowding. Each of these three CP-based through-space 2D spectra was superimposed on a sheared DP refocused *J*-INADEQUATE spectrum,^86^ for which the resonance assignment is available (Table S9).

The spatial proximities examined by a total of 594 cross peaks observed in this complete set of 2D spectra are summarized in Figure 5e and Table S9. Most algal carbohydrates are mobile since CP-based refocused INADEQUATE only reports 39% of the signals identified in the DP version. Analysis of the intramolecular cross peaks on the CP-based 2D spectra mostly allowed the identification of storage carbohydrates (associated with starch), together with a few other sugar units including xylose (Xyl or X), galactose (Gal or Ga), rhamnose (Rha or R), mannose (Man or M), and glucose (Glc or G) (Figure 5e and Table S9), indicating their partial involvement in the rigid phase. However, for galactose—the second most abundant carbohydrate after glucose—only one of the three types of galactoses stays partially rigid (unit 1), but this form accounts for 45% of all galactose signals (Table S8 and S9). The other units are much more mobile and most likely associated with galactolipids that are highly abundant in plastid membranes. Most other glycans are mobile, 20% of which are oligosaccharides or nonreducing end groups, identified mostly from their characteristic C1 resonances (92–99 ppm). The major findings will be discussed below according to the molecular types, starting with starch that behaves differently from other constituents of the microalgae, followed by cell-wall polysaccharides, and galactolipids.

### Distribution and Architecture of Cellular Carbohydrates

#### Crystalline Starch

**Figure 6:**
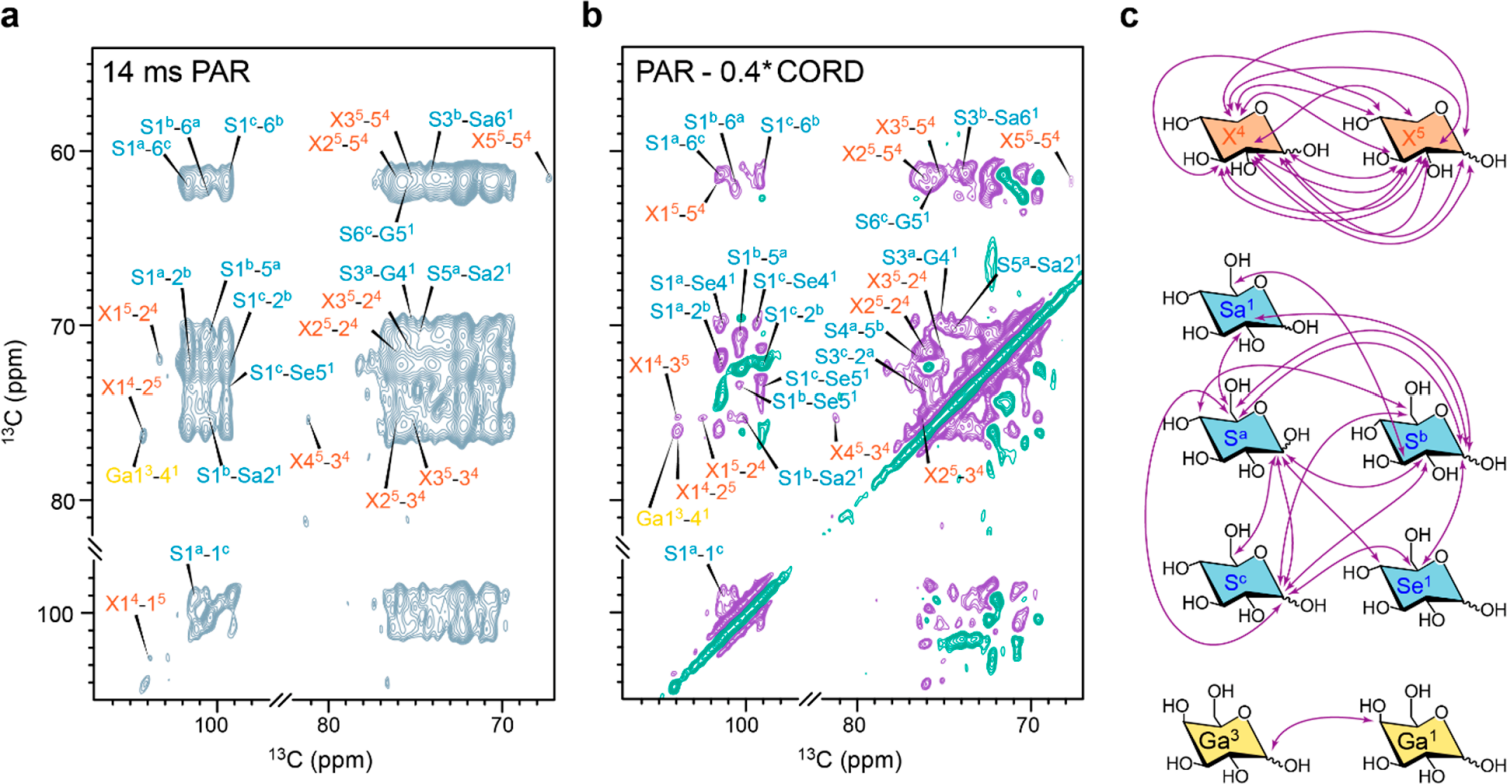
2D ^13^C–^13^C correlation spectra spotlighting inter-residue and intermolecular contacts. (a) 14 ms PAR spectrum with long-range intra- and intermolecular cross peaks labeled. (b) Difference spectrum obtained from two parental spectra (PAR and CORD) detecting only intermolecular or inter-residue contacts. (c) Representation of identified inter-residue contacts, mainly from xylose (top), starch (middle), and galactose units (bottom). Details of the observed cross peaks are listed in Table S9. All spectra were acquired with an 800 MHz spectrometer using 13.5 kHz MAS frequency, with 83 kHz TPPM for ^1^H decoupling.

The rigidity of starch might be ascribed to its crystallinity in *Parachlorella*’s reserve grain. Indeed, the PAR spectrum and the difference of two parental CORD and PAR spectra revealed strong inter-residue cross peaks within starch (Figure 6a,b), which confirmed the association of different glucose residues in this polymer. Such interactions happen between the anomeric carbons at 101 ppm (carbon 1 of starch unit a; S1^a^), 100 ppm (carbon 1 of starch unit b, S1^b^), and 99 ppm (carbon 1 of starch unit c; S1^c^) and several other carbons in other starch chains including starch-associated molecules (such as unit 1 of starch associated molecules; Sa^1^) and end-groups (such as unit 1 of nonreducing starch end-group, Se^1^) as summarized in Figure 6c and Table S9. The contacts between starch-associated molecules (small starch polymers or soluble fractions of starch) and the main spin systems of starch, as represented by the S1^b^–Sa2^1^, S5^a^–Sa2^1^, and S3^b^–Sa6^1^ cross peaks at 100.2–75.0, 75.0–70.5 and 74.0–61.4 ppm, respectively, revealed the close spatial proximity of these fractionated segments with the major starch domains. Contacts between end-groups and the crystalline part of starch, for example, the 99–73.5 ppm cross peak representing the S1^c^–Se5^1^ contact, confirmed a stable and dense organization.

The intensity of the starch-associated molecule with a C_1_ at 96.6 ppm is higher when using INEPT polarization transfer, as opposed to other starch-associated molecules previously assigned. This carbon could be associated with a smaller polymer decorating the surface of starch grains. Indeed, this spin system was partially assigned before in whole-cell samples using starchless strains but was absent in extracted starch.^90^ This can be a consequence of the starch extraction protocol that involves a centrifugation step selecting large or insoluble objects. This mobile starch-associated component is probably under 0.5 μm,^114^ which is the average size of extracted starch.

#### Dynamically Heterogeneous Polysaccharides in Parachlorella Cell Wall

Rigid glycans not related to starch, including galactose, xylose, and mannose units, were detected with the CP-based refocused INADEQUATE, RFDR, CORD, and PAR spectra (Table S9), suggesting a partially ordered structure or environment for these carbohydrates. These glycan components are known to be important for cell–cell interactions in microalgae.^61^ Moreover, glycans that are involved in establishing the cell-wall structural integrity through stable interactions with other glycans and protein units are also likely to be rigid. This is the case for several glucomannans present in other algae and plant cell walls.^93^ Indeed, according to their high C_1_ chemical shifts, two of the three assigned galactoses are probably associated with polysaccharide units of glycoproteins in the network that constitutes the cell wall.

Mannose exhibits a binary distribution in both the rigid and mobile phases. As it is one of the carbohydrates reported in *Chlorella* cell walls^115^ and involved in its N-glycosylation,^116^ we could expect a similar glycosylation scheme in the closely related *Parachlorella* cell wall. Therefore, the rigid mannose residues observed in CP-based 2D spectra might be close to the protein portion of the cell wall. More dynamic mannose residues were also identified using 1D INEPT and 2D refocused DP-INADEQUATE spectra, resolving the nonreducing mannose with a very low C_1_ chemical shift of 93 ppm (mannose unit 2). This observation suggests the presence of hydrated mannose at the cell-wall surface. It supports previous studies where *Chlorella* cell wall was shown to be mostly made of glucose and mannose,^117^ thus stressing the importance of these carbohydrates for polysaccharide interaction in the cell wall or between different cells as mobile signaling agent.^118^

**Figure 7:**
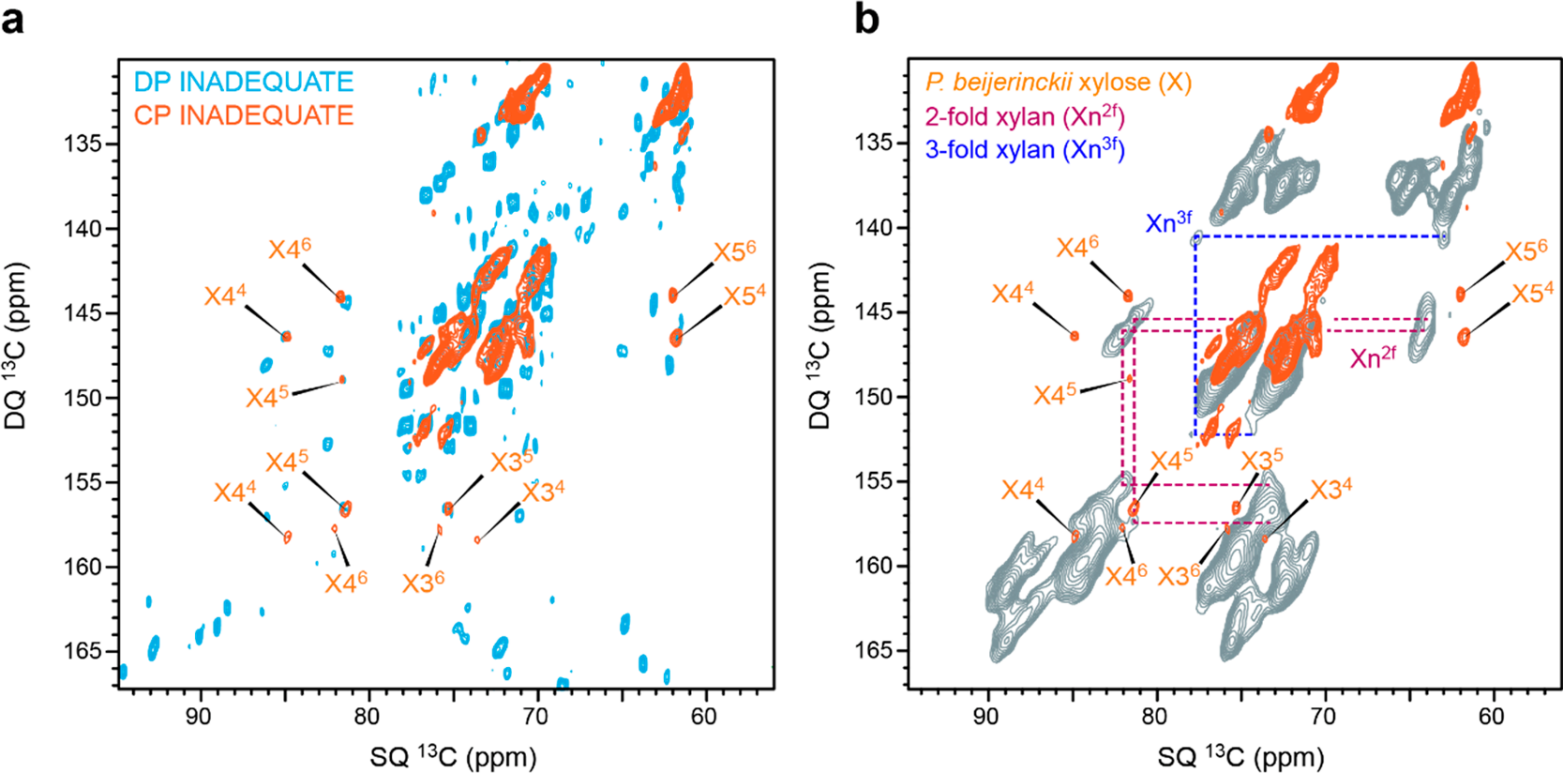
Comparison of algal xylose with plant xylan. (a) Overlay of refocused INADEQUATE spectra measured with CP (orange) and DP (cyan) on *P. beijerinckii* whole cells, with an 800 MHz NMR spectrometer under 13.5 kHz MAS, with 83 kHz TPPM decoupling. Subtypes 4, 5, and 6 of *P. beijerinckii* xyloses show signals in both CP- and DP-based spectra, revealing their distribution in both rigid and mobile domains. (b) The signals of these algal xyloses are more similar to the 2-fold xylan (Xn^2f^) observed in maize (gray) than to the 3-fold conformer (Xn^3f^) reported recently.^36^ The reference spectrum of maize xylan was acquired with a 600 MHz spectrometer using a 14 kHz MAS frequency with 83 kHz TPPM decoupling. This maize spectrum was adapted with permission from Kang et al.^36^ Copyright (2019) Nature (https://creativecommons.org/licenses/by/4.0/).

We were also able to identify preferential contacts between different xylose units, in particular, between types 4 and 5 (Figure 6c), supporting their involvement in stabilized linkages within this polymer that might be decorating the cell surface. Among the six types of xylose identified in *Parachlorella*, three forms (types 4–6) were simultaneously detected in both CP- and DP-based spectra (Figure 7a), revealing their heterogeneous dynamics, with coexisting rigid and mobile domains. Previously, algal xylose has been reported in other polysaccharide assemblies, for example, those involving xylan in 2- and 3-fold conformations (2 or 3 sugar units per helical turn),^119^ which were also found in plant cell walls.^72^ As a sensitive indicator of helical screw conformations, the C_4_ chemical shifts (80–84 ppm) of types 4–6 xyloses indicate that their structures are more like the 2-fold form (Figure 7b). In plants, the flat-ribbon structure of 2-fold xylan was typically considered as being enforced by the deposition of this hemicellulose on a flat surface (for example, cellulose microfibrils) and might be reverted to the more favorable 3-fold conformation in the absence of cellulose.^72^ Therefore, the observation of 2-fold-like xylose residues in the cellulose-deficient *Parachlorella CK-5* strain is counterintuitive. Since xyloses are mostly found in the algal cell walls and rarely in the cell, these units likely decorate the surface layers, with other molecules or mechanisms involved as a scaffold to stabilize this extended conformation.

#### Mobile Galactolipids and Other Glycan Units

Galactolipids have only one or two residues attached together, for MGDG/SQDG and DGDG, respectively (Figure 1c). They can be very abundant in photosynthetic organisms. For example, *Chlorella* nitrogen starvation can lead neutral lipids to reach 80% of the dry mass of the cell.^120^ One of the three assigned galactoses is mobile (galactose 2, Table S9) and has a uniquely small (upfield) C_1_ chemical shift at 98.4 ppm (galactose unit 2) revealed by the INEPT spectrum (Figure 2), indicative of its presence in mono- or oligosaccharides or nonreducing end groups, which are mostly found in galactolipids. This observation is consistent with previous galactolipid assignment in microalgae using ssNMR.^45^ Galactolipids are therefore readily detectable in whole cells, and peptide-membrane interaction studies in situ could be considered. Observation of rhamnose (21 ppm; unit 1, 2 and 3) and arabinose (108 ppm; units 1 and 2) in the INEPT spectrum also suggests their presence in oligosaccharides or end-groups decorating the surface of the cell wall or being involved in cell–cell interaction and signaling.

## Conclusions and Perspectives

Using *Parachlorella* microalgae, we showed that high-field ssNMR of ^13^C-labeled cells allows the detection, assignment, and quantification of glycans, and provides valuable structural and dynamic information in cells. With improved protocols, ssNMR quantification of glycan composition in intact cells becomes possible, although cross-validation with MS results is recommended. The most abundant carbohydrates in *Parachlorella* are glucose units from starch, followed by galactose from galactolipids, then the cell wall components. Most glycans in *Parachlorella beijerinckii CK-5* are highly dynamic, except for glucose in the crystalline starch and minor glycans probably associated with the cell wall.

*Parachlorella* species are frequently misnamed *Chlorella*, because these two algal taxons are very similar and mainly distinguished by fine genome differences. The high molecular mobility and the lack of cellulose in the current *Parachlorella* strain might explain, at least partially, why this microalga is easier to break down and digest than certain *Chlorella* strains that have stiff fibrillar cellulosic cell walls.^41^ Our data suggest that the very high content of starch and mobile carbohydrates might be determinant, making this type of alga a potential template for the design of new strains for bioenergy production, in addition to its potential use in bioremediation or as a food supplement already under investigation.^121^ Both noncellulosic *Chlorella* strains (such as several *Chlorella vulgaris* strains^122^) and cellulose-rich algal species (such as *Chlorella sorokiniana* strains^123^) are therefore of high interest for further investigation. Multiple strains have been designed to overproduce cellulose with the consideration that even cellulosic bioethanol should be useful, for example, as demonstrated on plant biomass.^124^ A better understanding of the interactions between cellulose and other cell wall components, and between different cellulose molecules, might help identify the key structural factors that have been limiting the accessibility of inner molecules and hindering the efficient utilization of microalgal biomass. Actually, in the primary cell walls of plants, the lateral interactions between cellulose microfibrils were found to be determinant to mechanical properties as revealed by coarse-grained molecular dynamics simulation.^125^ Since the structure of cellulose is known to differ in algae and higher plants, the structural function of cellulose in the organization of algal cell walls requires further assessment by comparing the cellulose-deficient strain with cellulose-rich species.

The difficulty in carbohydrate analysis necessitates the development of new software and methods, likely based on statistical analysis, to expedite glycan assignment according to the available chemical shifts, spin system, and potentially *J*-coupling patterns. Future work involving starchless or mutant strains removing certain carbohydrate and multinuclear labeling schemes could allow better identification of other glycans and their contacts with amino acids in glycoproteins. The approaches established here are extendable to a wide spectrum of microalgal species, such as *Chlamydomonas* and *Chlorella*, which have the potential of facilitating ongoing efforts in optimizing their use for biopharmaceutical, bioenergy, and nutraceutical industries.

## Supporting Information Available

Comparison of fresh and rehydrated algal cells (Figure S1); EI-MS data of ^13^C labeled *P. beijerinckii* glycans (Figure S2); Comparison of ^13^C–^13^C spectra from *P. beijerinckii* and other algae (Figure S3); Comparison of CP and DP-based INADEQUATE spectra (Figure S4); Representative signals of algal carbohydrates (Figure S5); Protein signals (Figure S6); Flowchart for glycan quantification (Figure S7); Calibration factor of starch content (Figure S8); Peak splitting (Figure S9); Glycosyl composition analysis (Table S1); Glycosyl linkage analysis (Table S2); SsNMR experiments and parameters (Table S3); Observed ^13^C chemical shifts (Table S4); Comparison with literature chemical shifts (Table S5); Tentative assignments of ambiguous spin pairs (Table S6); Quantification methods (Table S7); Intensities for quantification (Table S8); Identified internuclear correlations (Table S9) (PDF)

## Abbreviations

CORD: combined R2^υ^_n_-driven recoupling
CP: cross-polarization
DGDG: digalactosyldiacylglycerol
DP: direct polarization
DQ: double quantum
GC: gas chromatography
INADEQUATE: incredible natural abundance double quantum transfer experiment
INEPT: insensitive nuclei enhancement by polarization transfer
MAS: magic-angle spinning
MGDG: monogalactosyldiacylglycerol
MS: mass spectrometry
PAR: proton assisted recoupling
PMAA: partially methylated alditol acetates
RF: radio frequency
RFDR: radio frequency driven recoupling
SQ: single quantum
SQDG: sulfoquinovosyldiacylglycerol
ssNMR: solid-state NMR
TFA: trifluoroacetic acid.
