# Supplementary material for: Identification and Quantification of Glycans in Whole Cells: Architecture of Microalgal Polysaccharides Described by Solid-State Nuclear Magnetic Resonance
Source: J Am Chem Soc. 2021 Nov 4;143(46):19374–88. doi: 10.1021/jacs.1c07429 (PMC8630702; doi:10.1021/jacs.1c07429)
Supplement: Supplementary file 1 — ja1c07429_si_001.pdf [file ja1c07429_si_001.pdf]

Identification and Quantification of Glycans in Whole Cells: Architecture of Microalgal Polysaccharides Described by Solid-State Nuclear Magnetic Resonance

Alexandre Poulhazan[1], Malitha C. Dickwella Widanage[2], Artur Muszyński[3], Alexandre A. Arnold[1], Dror E. Warschawski[4], Parastoo Azadi[3], Isabelle Marcotte[1]*, Tuo Wang[2]*

[1] Department of Chemistry, University of Quebec at Montreal, Montreal, H2X 2J6, Canada

[2] Department of Chemistry, Louisiana State University, Baton Rouge, LA, USA

[3] Complex Carbohydrate Research Center, University of Georgia, Athens, GA 30602, USA

[4] Laboratoire des Biomolécules, LBM, CNRS UMR 7203, Sorbonne Université, École normale supérieure, PSL University, 75005 Paris, France

* Corresponding authors: tuowang@lsu.edu; marcotte.isabelle@uqam.ca

Table of Contents

Figure S1. Comparison of never-dried and rehydrated microalgal cells 3

Figure S2. Electron ionization mass spectra of ^{13}C labeled *P. beijerinckii* glycans 4

Figure S3. Comparison of *P. beijerinckii* and other algae 5

Figure S4. Comparison of DP and CP J-INADEQUATE spectra 6

Figure S5. Representative spin systems assigned using refocused INADEQUATE spectrum 7

Figure S6. Protein signals in *P. beijerinckii* cells 8

Figure S7. Flow chart for glycan quantification 9

Figure S8. Calibration factor for starch content 10

Figure S9. Peak splitting observed on carbohydrates in whole cells 11

Table S1. Glycosyl composition analysis of the neutral sugars in *P. beijerinckii*. 12

Table S2. ^{13}C-glycosyl linkages of uniformly ^{13}C labeled *P. beijerinckii* cells. 13

Table S3. MAS ssNMR experiments used for characterizing *P. beijerinckii* cells. 14

Table S4. ^{13}C chemical shifts of polysaccharides in *P. beijerinckii* CK-5 cells. 15

Table S5. Comparison of observed ^{13}C chemical shifts with literature values. 16

Table S6. Tentative assignment of ambiguous spin pairs. 20

Table S7. Details of the different methods used for quantification. 22

Table S8. ^{13}C raw integrals used for quantification of *P. beijerinckii* polysaccharides. 23

Table S9. List of correlations found in the different experiments. 27

References for glycan assignment 32

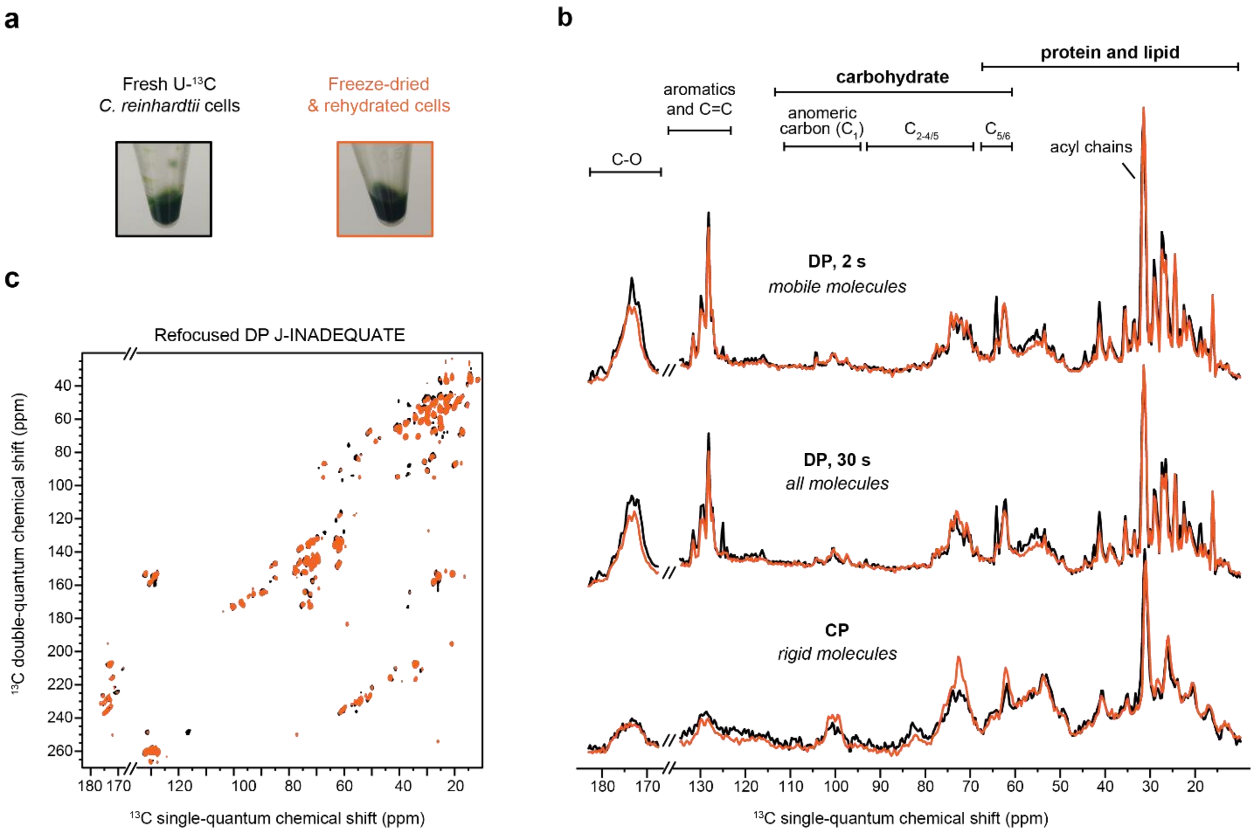

Figure S1. **Comparison of never-dried and rehydrated microalgal cells.** (**a**) Pictures of fresh and never-dried *C. reinhardtii* cells (left, black) and freeze-dried and then rehydrated cells (right, orange). (**b**) 1D ^{13}C spectra of fresh samples (black) and rehydrated materials (orange). From top to bottom are the DP spectrum with a 2 s recycle delay to detect mobile molecules, DP spectrum with a 30 s recycle delay for quantitative detection, and CP spectrum to select rigid components. Only relatively small changes were observed for the protein fraction and glycans, except for a slight increase of the starch signal in the CP spectrum, which can be due to biological variations. (**c**) 2D refocused DP J-INADEQUATE spectra largely retaining a comparable pattern after freeze-drying and rehydration.

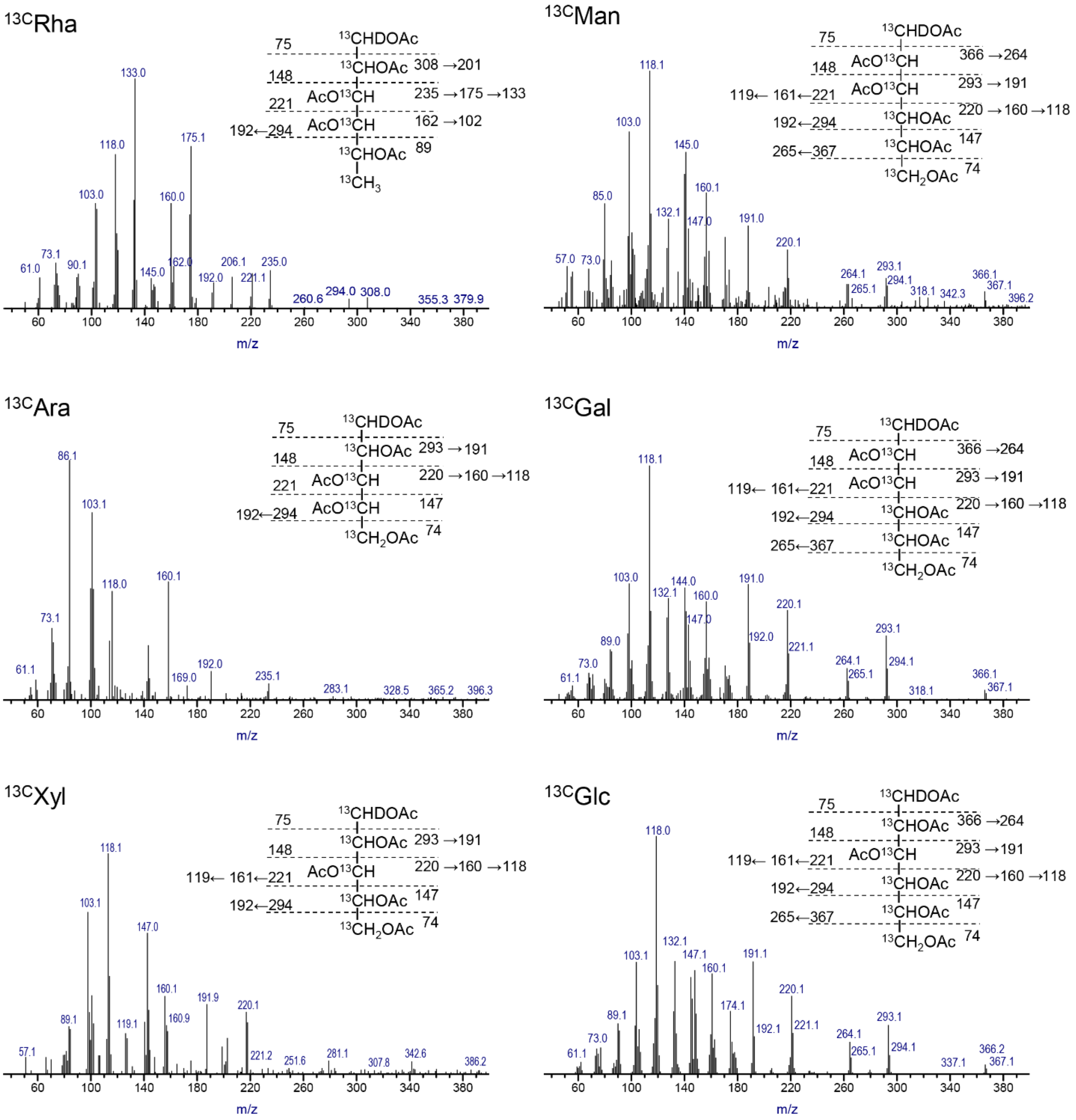

Figure S2. Electron ionization mass spectra (EI-MS). Proposed interpretation of the fragmentation patterns for alditol acetates of ^{13}C-labeled (~100%) neutral glycosyl residues identified in the *P. beijerinckii* cells are provided.

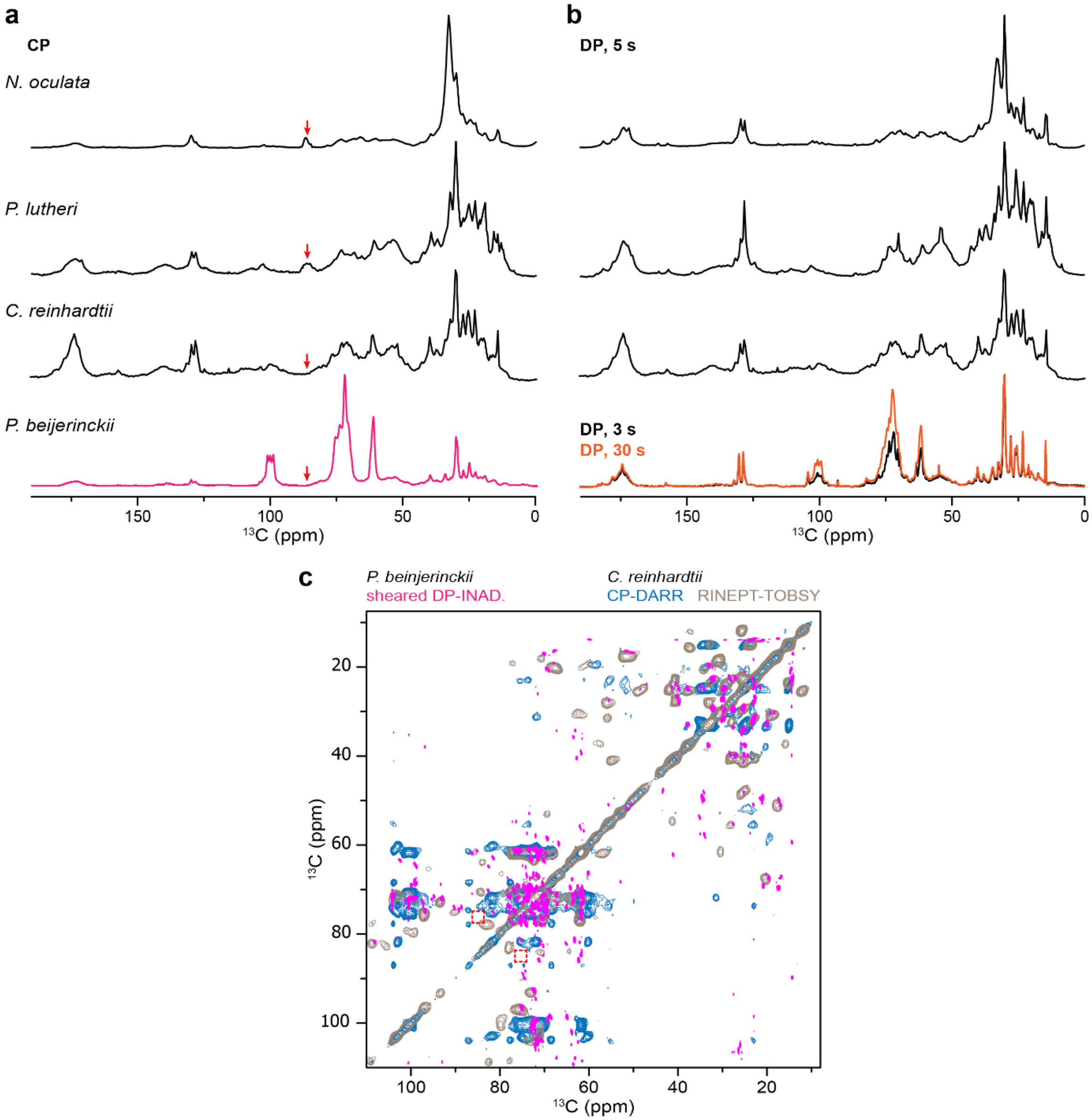

Figure S3. **Comparison of *P. beijerinckii* with other microalgae.** *P. beijerinckii* spectra were collected with an 800 MHz NMR spectrometer while the data of other three samples were adapted from Arnold *et al.*[1,2] obtained with a 400 MHz spectrometer. (**a**) 1D ^{13}C CP spectra of *P. beijerinckii* and other microalgae showing a different cellulose content (highlighted by arrows). *Nannochloropsis oculata* and *Pavlova lutheri* are cellulose-rich marine microalgae producing chrysolaminarin for energy storage, while *Chlamydomonas reinhardtii* has a glycoprotein-rich cell wall and produces starch as energy storage. (**b**) 1D ^{13}C DP spectra (with 3 and 30 s recycle delays) of *P. beijerinckii* and other microalgae (with a 5 s recycle delay) show a high content of carbohydrate components. (**c**) Partial similarity between *P. beijerinckii* (magenta) and *C. reinhardtii* (blue and grey for CP-DARR and RINEPT-TOBSY, respectively) carbohydrates as revealed by signal overlap in 2D ^{13}C-^{13}C correlation spectra. Dashed rectangles are expected C3/5-C4 correlation range for cellulose. Data of *N. oculata*, *P. lutheri*, and *C. reinhardtii* were adapted from Arnold *et al.*[1,2]. Copyright 2015 Elsevier and 2018 Springer Nature.

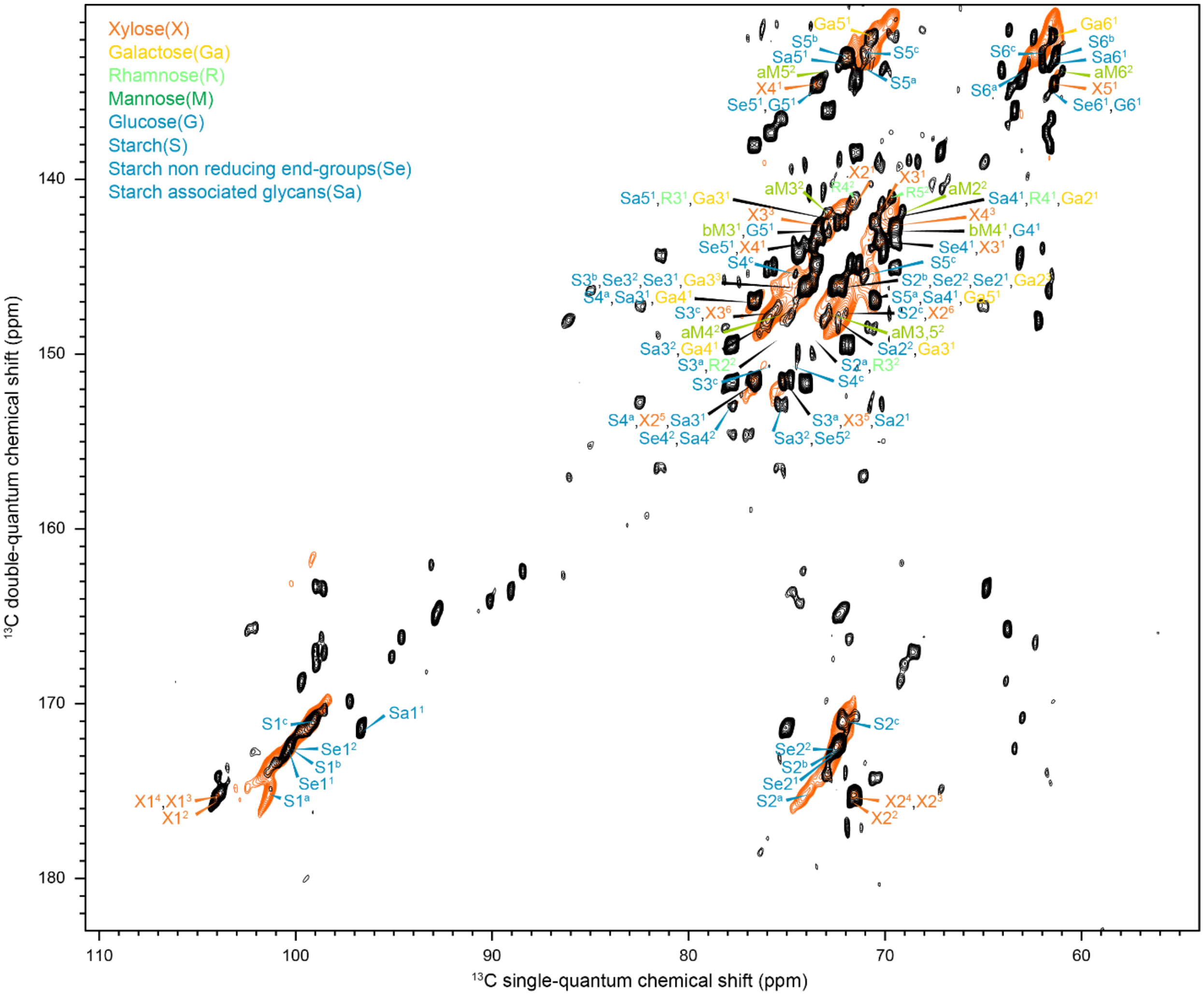

Figure S4. **Comparison of DP and CP J-INADEQUATE spectra.** The CP-based spectrum (orange) primarily shows rigid molecules, mostly from starch and xylose units. The DP-based spectrum (black) shows many more signals from different sugar units. The spectrum was acquired with an 800 MHz NMR and a 13.5 kHz MAS frequency.

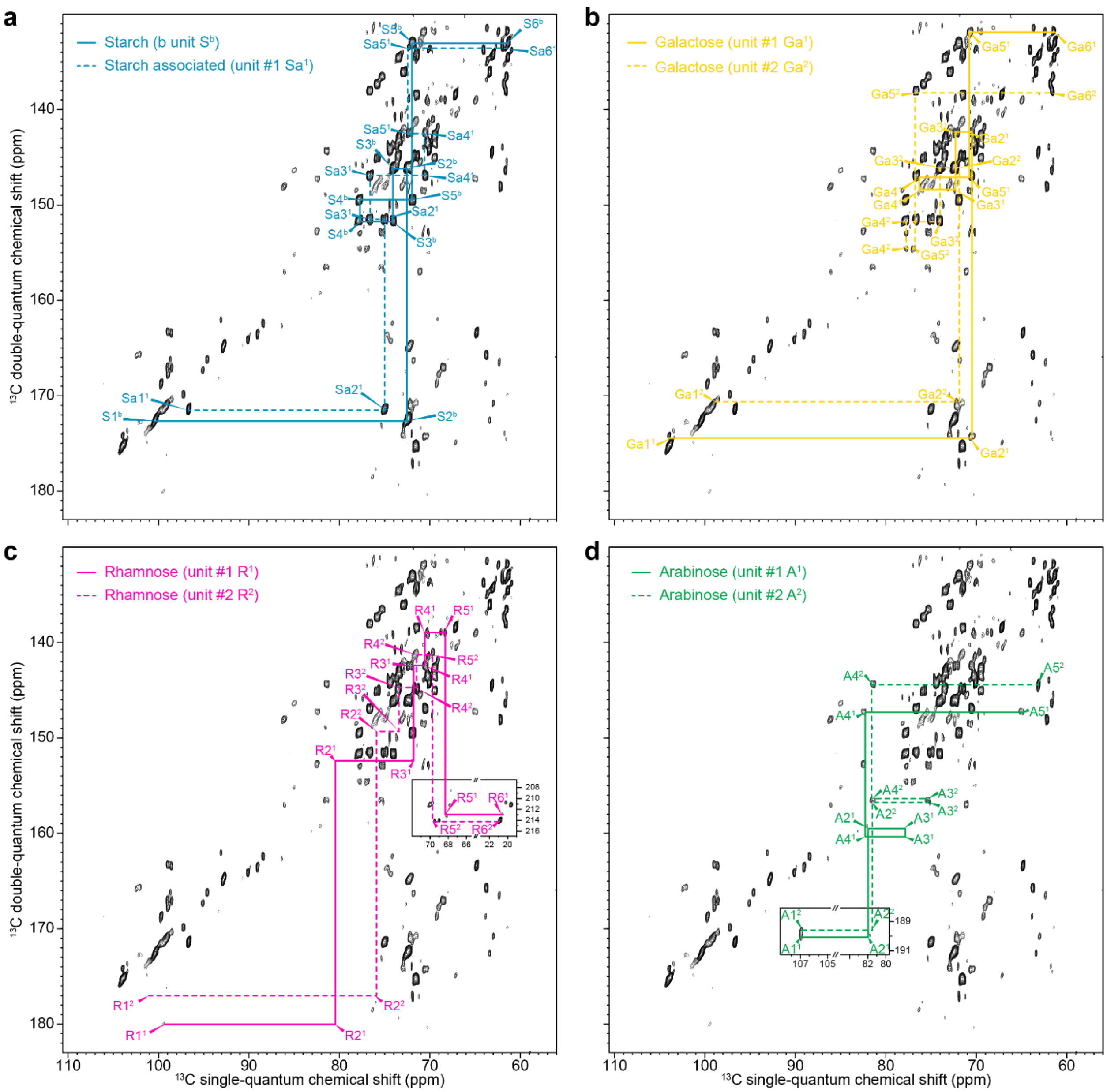

Figure S5. Representative spin systems assigned using refocused DP *J*-INADEQUATE. Complete carbon connectivity was shown for multiple *P. beijerinckii* carbohydrates, including (**a**) starch (including one starch-associated unit), (**b**) galactose, (**c**) rhamnose, and (**d**) arabinose. The spectrum was collected on with an 800 MHz NMR and a 13.5 kHz MAS frequency

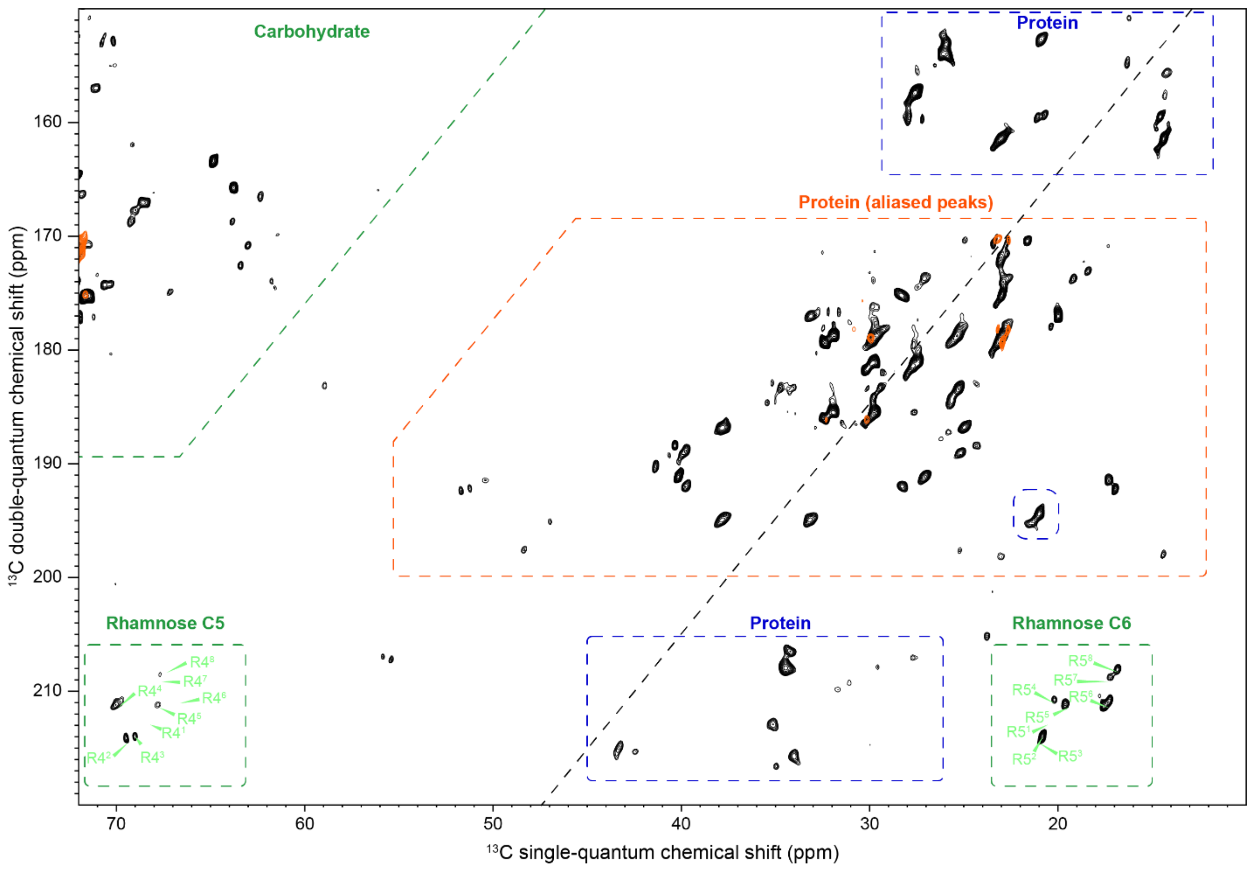

Figure S6. **Protein signals in *P. beijerinckii* cells.** Zoom of the aliased region of the refocused DP (black contours) and CP (orange contours)-based J-INADEQUATE. Some of the peaks are not aliased in this window and come from other amino acid residues (blue dashed lines). Most of the aliased peaks come from amino acids (orange region), but rhamnose residues (green region), with their specific low C_6 chemical shift, are also aliased. The black dashed line represents the hypothetical diagonal if this part of the spectrum was not aliased.

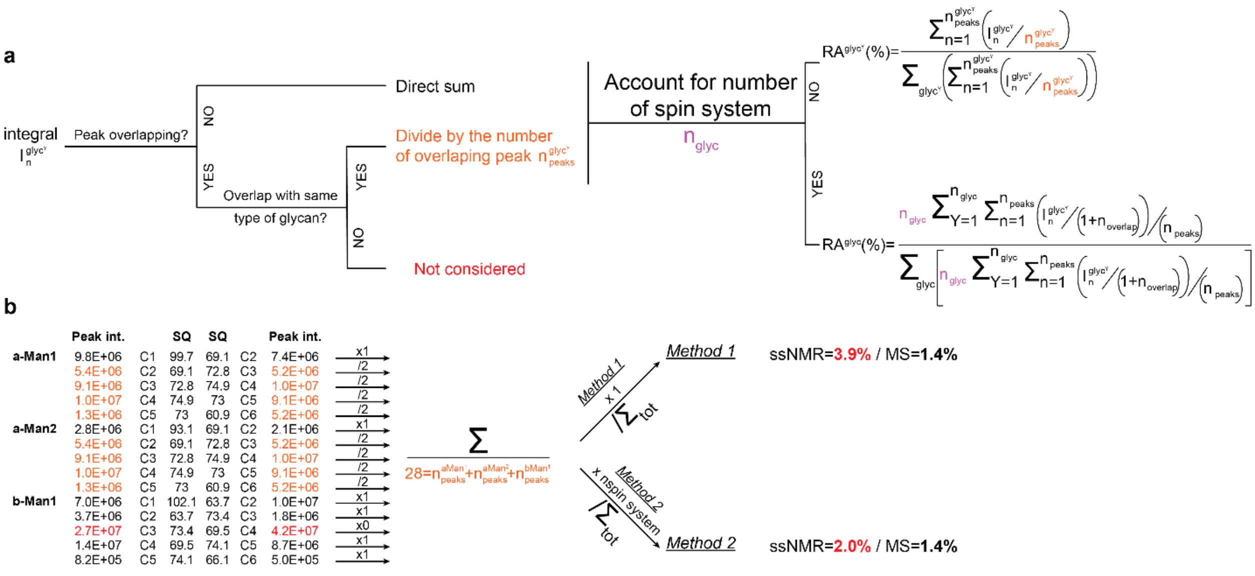

Figure S7. **Decision tree for peak integral from a refocused INADEQUATE spectrum.** (**a**) This chart allows the introduction of integral normalization to account for the number of peaks from the spectrum. (**b**) Representative example of the quantification of mannose units using both Methods 1 and 2 as described in this work, showing that method 2 leads to a better agreement with mass spectrometry.

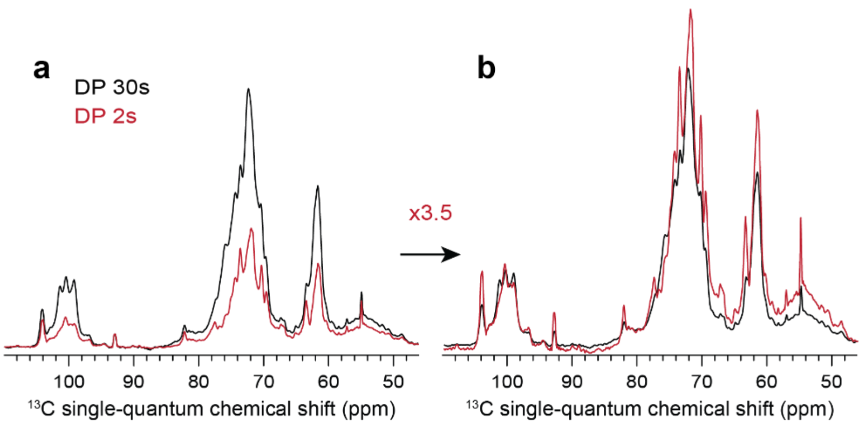

Figure S8. **Calibration factor for starch content.** After normalization by the number of scans, (**a**) the two ^{13}C DP spectra with recycling delays of 30 and 2 s are adjusted to (**b**) match the starch C1 intensity at 100 ppm. This gives the 3.5 correction factor to adjust starch intensity described in the main text and in **Figure 4c** and detailed in **Table S7**.

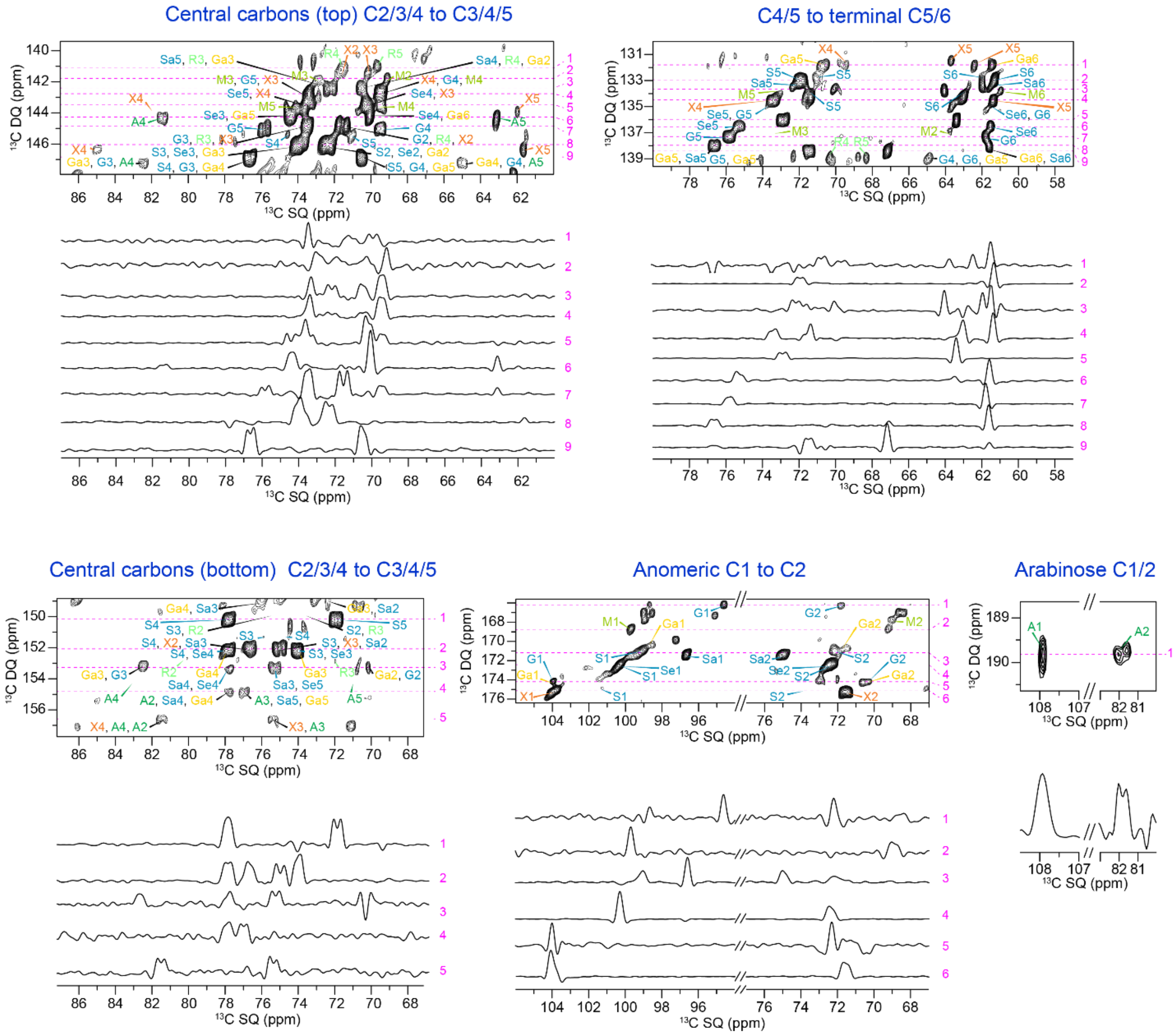

Figure S9. **Peak splitting observed on carbohydrates in whole cells.** The refocused DP *J*-INADEQUATE spectrum and the extracted cross sections are shown for different carbon sites. Doublets could be observed for most central carbons but not for the anomeric carbon (C1) or the terminal carbons (C5 or C6 depending on the sugar structure).

Table S1. Glycosyl composition analysis of the neutral sugars in *P. beijerinckii*. Results were obtained using uniformly ^{13}C-labeled cells and mass spectrometry. The glucose (Glc) line is in bold because it is the main species found, mainly due to high amount of starch in the cell.

^{13}C alditol	Relative mole (%)
Ara	0.8
Rha	3.7
Xyl	1.1
Man	1.4
Gal	8.2
**Glc (including starch)**	**84.8**

Table S2. ^{13}C-glycosyl linkages of uniformly ^{13}C labeled *P. beijerinckii* cells. Results were determined by gas chromatography-mass spectrometry (GC-MS). The percentages given below are the peak area from the EI detector response (%). The species in bold are the most abundant linkages mostly associated with starch molecules.

^{13}C linkage	Relative EI detector response (%)
t-Araf	1.19
1,2/1,3-Rhap	2.85
t-Xylp	1.06
t-Manp	0.46
t-Galf	0.77
t-Galp	0.81
1,6-Galp	4.84
1,3-Galp	3.85
1,3/1,6-Galp	1.21
1,3/1,4/1,6-Galp	3.63
t-Glcp	2.17
t-Glcf	0.51
**1,4-Glcp**	**44.93**
**1,3/1,4-Glcp**	**23.10**
1,2/1,3-Glcp	1.74
**1,4/1,6-Glcp**	**6.89**

Table S3. **MAS ssNMR experiments used for characterizing *P. beijerinckii* cells.** 1D spectra allowed to excite different regions of the microalgal cells based on their dynamics. To obtain a quantitative spectrum, we used direct pulse (DP) experiments with a 30 s recycling delay. Using cross-polarization (CP), the most rigid molecules were selected. With DP and a shorter recycling delay of 2 s, rigid molecules were suppressed from the spectrum, and with Insensitive Nuclei Enhanced by Polarization Transfer (INEPT), the most mobile molecules were selected. The SQ 2D spectra were obtained using 14 ms PAR (Proton-Assisted Recoupling), 1.5 ms RFDR (Radio-Frequency Driven Recoupling), and 53 ms CORD (COmbined $R2^{v}_{n}$-Driven recoupling). For glycan assignment, DQ 2D spectra were used, either DP or CP-INADEQUATE (Incredible Natural Abundance DoublE QUAntum Transfer Experiment).

	Experiment	Total time (h)	Recyc. Delay (s)	n. scans	n. point	n. increment	Aq. (ms)	indirect Aq. (ms)
1D	DP	0.1	2	128	4096	1	28.7	
	DP	0.5	30	64	4096	1	28.7	
	CP	0.1	2	128	2400	1	16.8	
	INEPT	0.2	4	128	4096	1	28.7	
2D SQ-SQ	PAR	6	2	64	1920	172	14.3	4.5
	RFDR	3	2	16	2500	320	18.7	8
	CORD	5	2	16	2400	560	17.9	7.3
2D DQ-SQ	DP-INADEQUATE	3	2	16	2400	350	17.9	7
	CP-INADEQUATE	1.7	2	16	2400	200	17.9	6.5

Table S4. **^{13}C chemical shifts of polysaccharides in *P. beijerinckii CK-5* cells.** Underlined values could be associated with other glycans due to chemical shift ambiguity when comparing to literature values. (/) is used for unidentified carbons. All chemical shifts will be deposited on the CCMRD database, with the original Bruker dataset available upon request.

Sugar Type	C1	C2	C3	C4	C5	C6	Ac	Met
**Starch associated units**								
starch a	101.3	73.9	75.4	76.6	70.5	62.7		
starch b	100.2	72.4	74	77.7	71.8	61.3		
starch c	99	72	76	74.7	70.9	62		
starch-as-Glc1	96.6	75	76.6	70.4	72	61.4		
starch-as-Glc2	92.7	72.4	75.4	77.6	76.8	61.5		
starch-end1	100.3	72.3	73.7	70.1	73.5	61.3		
starch-end2	100.2	72.4	74	77.7	75.1	61.4		
**Glucose units**								
Glucan 1	/	/	/	69.4	75.7	61.7		
Glucan 2	104	70.1	82.4	64.8	74.1	64.8		
Glucan 3	102.7	73.7	67.1	/	/	/		
Glucose1	94.6	71.7	73.4	69.4	73.4	61.4		
**Arabinose units**								
Ara1	107.9	81.7	77.8	82.2	64.7			
Ara2	108	81.7	75.2	81.3	63.1			
Ara3	/	89	74.8	81.6	63			
Ara4	/	78	77	83	71			
**Galactose units**								
Gal1	104	70.3	72.2	76.3	70.5	61.3		
Gal2	98.4	72.2	73.9	77.9	76.7	61.6		
Gal3	104	70.2	82.4	65.1	74	70		
**Mannose units**								
a-Man1	99.7	69.1	72.8	74.9	73	60.9		
a-Man2	93.1	69.1	72.8	74.9	73	60.9		
b-Man1	102.1	63.7	73.4	69.5	74.1	66.1		
**Rhamnose units**								
Rha1	99.6	80.3	72	70.4	68.2	20.5		
Rha2	101.1	75.8	73.2	71.6	69.4	20.9		
Rha3	/	/	/	/	69	20.8		
Rha4	/	/	/	/	69.9	17.3		
Rha5	/	/	/	/	67.7	19.5		
Rha6	/	/	/	/	66.6	20.2		
Rha7	/	/	/	/	67.6	17.3		
Rha8	/	/	/	/	67.3	16.8		
**Xylose units**								
Xyl1	103.9	71.5	*70.1*	73.4	61.3			
Xyl2	104	71.6	73.2	67.6	63.6			
Xyl3	103.9	71.5	73.4	69.3	62.4			
Xyl-2fold4	103.9	71.5	73.5	84.9	61.6			
xyl-2fold5	102.5	76.2	75.3	81.3	67.5			
Xyl-2fold6	104.8	72	75.8	82	61.8			
**Acetyl-methyl groups**								
Ac-Met1							21.2	174.4
Ac-Met2							20.8	173.8

Table S5. Comparison of observed ^{13}C chemical shifts with literature values. The values obtained using ^{13}C-labeled *P. beijerinckii* cells were compared to literature values found in the CCMRD database. References cited here are given in a dedicated section below. Values with the best match with the literature are given in bold. Values of the $^{1}J_{CC}$ couplings deduced from deconvolution of slices from the refocused DP-INADEQUATE spectrum are given in a dedicated column. The "s" indicates the peak that is not splitted and is a singlet.

Glycan units		SQ	SQ		$^{1}J_{CC}$ (Hz)	reference 1		reference 2		reference 3	
**starch a**						Poulhazan 2018[3]		Rondeau-M. 2006[4]		Poulhazan 2018[3]	
	C1	101.3	73.9	C2	s/76.0	100.6	**72.5**	100.2	72.1	**101.8**	**73.6**
	C2	73.9	75.4	C3	78.2/65.2	**72.5**	74.2	72.1	**75.1**		
	C3	75.4	76.6	C4	79.6/89.6	74.2	**77.9**	**75.1**	74.3		
	C4	76.6	70.5	C5	83.2/65.2	**77.9**	71.9	74.3	**70.2**		
	C5	70.5	62.7	C6	72.4/s	71.9	61.6	**70.2**	**61.3**		
**starch b**						Paris 1999[5]		Poulhazan 2018[3]			
	C1	100.2	72.4	C2	s/78.3	**100.4**		**100.6**	**72.5**		
	C2	72.4	74.0	C3	82.4/58.3			**72.5**	**74.2**		
	C3	74.0	77.7	C4	58.3/65.6			**74.2**	**77.9**		
	C4	77.7	71.8	C5	65.6/65.6			**77.9**	**71.9**		
	C5	71.8	61.3	C6	66.3/s			**71.9**	**61.6**		
**starch c**						Paris 1999[5]		Rondeau-M. 2006[4]		Poulhazan 2018[3]	
	C1	99.0	72.0	C2	s/71.6	**99.4**		100.2	72.1	**99.1**	**71.9**
	C2	72.0	76.0	C3	76/57.9			**72.1**	**75.1**		
	C3	76.0	74.7	C4	50.7/49.8			**75.1**	**74.3**		
	C4	74.7	70.9	C5	67.5/64.9			**74.3**	**70.2**		
	C5	70.9	62.0	C6	68.8/s			**70.2**	**61.3**		
**starch-as-Glc1**						Poulhazan 2018[3]		Poulhazan 2018[3]		Bradbury 1984[6]	
	C1	96.6	75.0	C2	s/76.7	**96.7**	**74.9**	100.5	72.3	92.9	72.5
	C2	75.0	76.6	C3	66.3/55.7			72.3	73.7	72.5	**73.8**
	C3	76.6	70.4	C4	83.2/65.2			73.7	**70.4**	**73.8**	**70.6**
	C4	70.4	72.0	C5	76/54.3			**70.4**	**73.7**	**70.6**	**72.3**
	C5	72.0	61.4	C6	61.5/s			**73.7**	**61.1**	**72.3**	**61.6**
**starch-as-Glc2**						Poulhazan 2018[3]		Roubroeks 2001[7]			
	C1	92.7	72.4	C2	s	**92.7**	**72.4**				
	C2	72.4	75.4	C3		**72.4**	**74.9**	73.8	75		
	C3	75.4	77.6	C4	57.9/83.2	**74.9**	**76.9**	**75**	**79.4**		
	C4	77.6	76.8	C5	65.2/65.2	**76.9**	70.4	**79.4**	**76.8**		
	C5	76.8	61.5	C6	68.8/s	70.4	**61.4**	**76.8**	**60.9**		
**starch-end1**						Poulhazan 2018[3]					
	C1	100.3	72.3	C2	s	100.5	72.3				
	C2	72.3	73.7	C3	86.9/72.4	72.3	73.7				
	C3	73.7	70.1	C4	68.8/57.9	73.7	70.4				
	C4	70.1	73.5	C5	57.9/61.5	**70.4**	**73.7**				
	C5	73.5	61.3	C6	72.4/s	**73.7**	**61.1**				
**starch-end2**											
	C1	100.2	72.4	C2	*N.A*/s						
	C2	72.4	74.0	C3	86.9/72.4						
	C3	74.0	77.7	C4	57.9/83.2	Rondeau-M. 2008[8]					
	C4	77.9	75.1	C5	N.A/N.A	77.9	73.7				
	C5	75.1	61.4	C6	61.5/s	73.7	61.9				
**Glucan 1**						Arnold 2015[9]					

	C1	/	/	C2	/	102.7	73.8		
	C2	/	/	C3	/	73.8	84.4		
	C3	/	/	C4	/	84.4	68.3		
	C4	69.4	75.7	C5	54.3/61.5	**68.3**	**75.9**		
	C5	75.7	61.7	C6	65.2/s	**75.9**	**60.9**		
**Glucan 2**						*Roubroeks 2001*[7]		*Pfeffer 1984*[10]	
	C1	104.0	70.1	C2	s/65.2	**103.1**	**73.7**	92.9	**70.5**
	C2	70.1	82.4	C3	57.9/37.9	**73.7**	**85.1**	**70.5**	73
	C3	82.4	64.8	C4	65.2/72.4	**85.1**	**68.9**	73	72.6
	C4	64.8	74.1	C5	N.A/N.A	**68.9**	76.4	72.6	**71.6**
	C5	74.1	64.8	C6	54.3/s	76.4	61	**71.6**	**63.7**
**Glucan3**						*Laguri 2018*[11]			
	C1	102.7	73.7	C2	s/61.3	103.3	74.3		
	C2	73.7	67.1	C3	32.6/43.4	74.3	70.8		
**Glucose1**						*Gorin 1975*[12]		*Bradbury 1984*[6]	
	C1	94.6	71.7	C2	s	92.7	**72.1**	92.9	**72.5**
	C2	71.7	73.4	C3	72.4/76	**72.1**	**73.4**	**72.5**	**73.8**
	C3	73.4	69.4	C4	N.A/N.A	**73.4**	**70.4**	**73.8**	**70.6**
	C4	69.4	73.4	C5	83.2/65.2	**70.4**	**72.1**	**70.6**	**72.3**
	C5	73.4	61.4	C6	72.4/s	**72.1**	**61.3**	**72.3**	**61.6**
**Ara1**						*Rondeau-M. 2008*[8]		*Wang 2014*[13]	
	C1	107.9	81.7	C2	s/N.A	**108.6**	**81.9**	110	**82.2**
	C2	81.7	77.8	C3	43.9/54.5	**81.9**	**78.1**	**82.2**	77.8
	C3	77.8	82.2	C4	43.9/54.5	**78.1**	**83.6**	77.8	**83**
	C4	82.2	64.7	C5	65.2/s	**83.6**	68	**83**	**64**
**Ara2**						*Rondeau-M. 2008*[8]			
	C1	108.0	81.7	C2	s/N.A	**108.6**	**81.9**	110	**82.2**
	C2	81.7	75.2	C3	68.8/76.0	**81.9**	78.1	**82.2**	77.8
	C3	75.2	81.3	C4	68.8/76.0	78.1	**83.6**	77.8	**83**
	C4	81.3	63.1	C5	79.6/s	**83.6**	68	**83**	**64**
**Ara3**						*Wang 2014*[13]		*Arnold 2015*[9]	
	C1	/	/	C2		107.3	87.8	103.2	77.5
	C2	89	74.8	C3	N.A/N.A	**87.8**	**72.6**	77.5	75.7
	C3	74.8	81.6	C4	68.8/76.0			**75.7**	**83.1**
	C4	81.6	63	C5	79.6/s			**83.1**	**64.2**
**Ara4**						*Kang 2018*[14]		*Arnold 2015*[9]	
	C1	/	/	C2	s/N.A	107.6	81.9	103.2	77.5
	C2	78	77	C3	65.2/65.2	81.9	**77.1**	**77.5**	**75.7**
	C3	77	83	C4	43.9/54.5	**77.1**	**83.3**	**75.7**	83.1
	C4	83	71	C5	N.A/s	**83.3**	**69.9**	83.1	64.2
**Gal1**						*Dick-Perez 2011*[15]		*Lundborg 2011*[16]	
	C1	104	70.3	C2	s/65.2	**105**	**72.6**	96.5	**69.6**
	C2	70.3	72.2	C3	76/54.3	**72.6**	**74.3**	**69.6**	70.1
	C3	72.2	76.3	C4	79.6/50.7	**74.3**	**78.4**	70.1	**77.6**
	C4	76.3	70.5	C5	83.2/65.2	**78.4**	75.2	**77.6**	**71.5**
	C5	70.5	61.3	C6	72.1/s	75.2	61.7	**71.5**	**61.1**
**Gal2**						*Wang 2014*[13]		*Habibi 2004*[17]	
	C1	98.4	72.2	C2	/	105	72.5	105.2	72.7
	C2	72.2	73.9	C3	86.9/72.4	**72.5**	**74.4**	**72.7**	**74.4**
	C3	73.9	77.9	C4	N.A/N.A	**74.4**	**78.4**	**74.4**	**78.5**
	C4	77.9	76.7	C5	65.2/65.2	**78.4**	**75.3**	**78.5**	**75.3**
	C5	76.7	61.6	C6	68.8/s	**75.3**	**61.7**	**75.3**	**61.9**
**Gal3**						*Tan 2010*[18]			

	C1	104	70.2	C2	s/65.2	**103.9**	**70**				
	C2	70.3	82.4	C3	57.9/37.9	**70**	**81.9**				
	C3	82.4	65.1	C4	65.2/72.4	**81.9**	**68.5**				
	C4	65.1	74	C5	N.A/N.A	**68.5**	**72.9**				
	C5	74	70	C6	68.8/s	**72.9**	**70.1**				
**a-Man1**						*Heux 2005*[19]		*Leung 2004*[20]		*Gorin 1975*[12]	
	C1	99.7	69.1	C2	s/54.8	**100.6**		**102.2**	**72.1**	**95**	**71.7**
	C2	69.1	72.8	C3	N.A/N.A			**72.1**	**73.5**	**71.7**	**71.3**
	C3	72.8	74.9	C4	N.A/N.A		**76.9**	**73.5**	**78.5**	**71.3**	68
	C4	74.9	73	C5	N.A/N.A	**76.9**		**78.5**	77.7	68	**73.4**
	C5	73	60.9	C6	N.A/s		**60.9**	77.7	**62.5**	**73.4**	**62.1**
**a-Man2**						*Lundborg 2011*[16]					
	C1	93.1	69.1	C2	s/N.A	94.9	71.2				
	C2	69.1	72.8	C3	61.5/54.3						
	C3	72.8	74.9	C4	61.5/65.2						
	C4	74.9	73	C5	61.5/65.2						
	C5	73	60.9	C6	54.3/s						
**b-Man1**						*Gorin 1975*[12]		*Lundborg 2011*[16]			
	C1	102.1	*63.7*	C2	s/61.3	**101.9**	71.2	94.9	71.2		
	C2	*63.7*	73.4	C3	57.9/56.8	71.2	**71.8**	71.2	79.3		
	C3	73.4	69.5	C4	54.3/61.5	**71.8**	**68**	79.3	**66.9**		
	C4	69.5	74.1	C5	50.7/56.4	**68**	**73.7**	**66.9**	**71.9**		
	C5	74.1	66.1	C6	N.A/s	**73.7**	62.1	**71.9**	**66.8**		
**Rha1**						*Habibi 2004*[17]					
	C1	99.6	80.3	C2	30.7/s	**101.9**	**79.3**				
	C2	80.3	72	C3	N.A/N.A	**79.3**	**73.7**				
	C3	72	70.4	C4	76/54.3	**73.7**	**71.1**				
	C4	70.4	68.2	C5	N.A/N.A	**71.1**	**70.4**				
	C5	68.2	20.5	C6	83.2/s	**70.4**	**17.8**				
**Rha2**						*Habibi 2004*[17]					
	C1	101.1	75.8	C2	41.6/s	**99.2**	**77.5**	94.6	72.4		
	C2	75.8	73.2	C3	N.A/N.A	77.5	70.2	**72.4**	**73.8**		
	C3	73.2	71.6	C4	72.4/76	70.2	**71.3**	**73.8**	**72.9**		
	C4	71.6	69.4	C5	64.2/54.3	**71.3**	**69.8**	**72.9**	**73.1**		
	C5	69.4	20.9	C6	N.A/s	**69.8**	**17.5**	**73.1**	**18**		
**Rha3**						*Bradbury 1984*[6]					
	C5	69	20.8	C6	41.6/s	**69.4**	**18**				
**Rha4**						*Phyo 2017b*[21]					
	C5	69.9	17.3	C6	81.1/s	69.7	17.3				
**Rha5**						*Habibi 2004*[17]					
	C5	67.7	19.5	C6	65.7/s	67.8	17.5				
**Rha6**						*Phyo 2017a*[22]					
	C5	66.6	20.2	C6	20.2/s	68.7	17.8				
**Rha7**						*Phyo 2017a*[22]					
	C5	67.6	17.3	C6	37.3/s	67.9	16.8				
**Rha8**						*Habibi 2004*[17]					
	C5	67.3	16.8	C6	37.3/s	67.8	17.5				
**Xyl1**						*Kang 2019*[23]		*Wang 2014*[13]			
	C1	103.9	71.5	C2	s/N.A	105.1	**72.5**	**102.4**	**73.9**		
	C2	71.5	*70.1*	C3	61.5/57	**72.5**	73.5	**73.9**	76.7		
	C3	*70.1*	73.4	C4	57.9/61.5	73.5	82.3	76.7	**74.6**		
	C4	73.4	61.3	C5	72.4/s	82.3	64.6	**74.6**	**63.6**		
**Xyl2**						*Kang 2019*[23]		*Wang 2014*[13]			

	C1	104	71.6	C2	s/83.3	105.1	**72.5**	**102.4**	**73.9**	
	C2	71.6	73.2	C3	72.4/76	**72.5**	73.5	**73.9**	76.7	
	C3	73.2	67.6	C4	27/47.9	73.5	82.3	76.7	**70.2**	
	C4	67.6	63.6	C5	N.A/s	82.3	**64.6**	**70.2**	**66**	
**Xyl3**						*Kang 2019*[23]		*Wang 2014*[13]		
	C1	103.9	71.5	C2	s/83.3	**105.1**	**72.5**	**102.4**	73.9	
	C2	71.5	73.4	C3	72.4/76	**72.5**	**73.5**	73.9	76.7	
	C3	73.4	69.3	C4	54.3/61.5	**73.5**	82.3	76.7	**70.2**	
	C4	69.3	62.4	C5	83.2/s	82.3	**64.6**	**70.2**	**66**	
**Xyl-2fold4**						*Kang 2019*[23]				
	C1	103.9	71.5	C2	s/83.3	**105.1**	**72.5**			
	C2	71.5	73.5	C3	72.4/76	**72.5**	73.5			
	C3	73.5	84.9	C4	N.A/N.A	73.5	**82.3**			
	C4	84.9	61.6	C5	61.5/s	**82.3**	**64.6**			
**Xyl-2fold5**						*Vignon 1998*[24]				
	C1	102.5	76.2	C2	s/50.4	**103**	**74**			
	C2	76.2	75.3	C3	66.3/55.7	**74**	75			
	C3	75.3	81.3	C4	68.8/76.0	75	**77.8**			
	C4	81.3	67.5	C5	N.A/s	**77.8**	**64.1**			
**Xyl-2fold6**						*Kang 2019*[23]		*Vignon 1998*[24]		
	C1	104.8	72	C2	s/N.A	**105.1**	**72.5**	98.8	72.6	
	C2	72	75.8	C3	76/57.9	**72.5**	73.5	72.6	**78.1**	
	C3	75.8	82	C4	53.3/54.3	73.5	**82.3**	**78.1**	**83.5**	
	C4	82	61.8	C5	79.6/s	**82.3**	**64.6**	**83.5**	73.6	
**Ac-Met1**										
	Met1	174.4	21.2	Ac1	52.5/65.7					
**Ac-Met2**										
	Met2	173.8	20.8	Ac2	55.8/70.3					

Table S6. Tentative assignment of ambiguous ^{13}C chemical shifts or spin systems. Not all spin pairs have been assigned in *P. beijerinckii* and reported in Table S4 and S5. Nevertheless, these unknown resonances are still reported below, and some are tentatively assigned and compared to literature values from the CCMRD database. The values with a best match with the literature are given in bold. The "s" indicate the peaks that are not splitted and are singlet.

Glycan units		SQ	SQ		$^1J_{CC}$ (Hz)	reference 1		reference 2		reference 3	
**Unk1 - Gal?**						Phyo 2017a[22]		Hantus 1997[25]			
	C1	98.7	64.9	C2	s/N.A	105.2	72.8	**99.6**	**69.6**		
	C2	64.9	74	C3	N.A/N.A	72.8	**74.3**	**69.6**	70.2		
	C3	74	77.9	C4	N.A/N.A	**74.3**	**78.5**	70.2	70		
	C4	77.9	70.7	C5	N.A/N.A	**78.5**	75.3	70	**71.5**		
	C5	70.7	61.3	C6	s/N.A.	75.3	**61.7**	**71.5**	**62**		
**Unk2 - Gal?**						Phyo 2017a[22]		Rondeau-M. 2008[8]		Gorin 1975[12]	
	C1	95.1	72.3	C2	s/N.A.	105.2	72.8	105.5	72.9	**93.6**	**69.8**
	C2	72.3	75.6	C3	N.A/N.A	**72.8**	**74.3**	72.9	**74.4**	69.8	70.6
	C3	75.6	77.5	C4	N.A/N.A	**74.3**	**78.5**	**74.4**	**78.8**	**70.6**	**70.6**
	C4	77.5	77.3	C5	N.A/N.A	**78.5**	75.3	**78.8**	**75.7**	70.6	71.7
	C5	77.3	68.3	C6	s/N.A.	75.3	61.7	**75.7**	61.6	71.7	62.5
**Unk3 - Gal?**						Hantus 1997[25]		Arnold 2015[9]			
	C1	88.4	74.1	C2	s/N.A.	99.6	69.6	103.4	**73**		
	C2	74.1	69.5	C3	N.A/N.A	69.6	70.2	**73**	**71.1**		
	C3	69.5	75.6	C4	N.A/N.A	70.2	70	**71.1**	**75.3**		
	C4	75.6	72.1	C5	N.A/N.A	70	71.5	**75.3**	**69.7**		
	C5	72.1	61.3	C6	s/N.A.	71.5	62				
**Unk4 - Ara?**						Wang 2014[13]		Phyo 2017a[22]			
	C3	71.0	86.1	C4	N.A/N.A	72.6	85.7	84.9	62.1		
	C4	86.1	62.1	C5	N.A/N.A						
**Unk5 - Met-Ara?**						Gorin 1975[12]					
	C1	102.2	71	C2	s/N.A.	101	69.4				
	C2	71.2	67.2	C3	N.A/N.A	69.4	68.9				
	C3	67	71.6	C4	N.A/N.A	68.9	70				
	C4	71.3	63	C5	N.A/N.A	70	63.8				
**Unk6 - Ara?**						Wang 2014[13]					
	C1	/	/	C2	s/N.A.	110	82.2				
	C2	82	76.8	C3	N.A/N.A	82.2	77.8				
	C3	76.8	82	C4	N.A/N.A	77.8	83				
	C4	82	63.2	C5	N.A/N.A	83	64				
**Unk7 - Ara?**						Wang 2014[13]					
	C1	/	/	C2	s/N.A.	107.3	87.8				
	C2	62.1	73.6	C3	N.A/N.A	87.8	72.6				
	C3	73.6	83.7	C4	N.A/N.A	72.6	85.7				
	C4	83.7	61.5	C5	N.A/N.A	85.7	62.4				
**Unk8 - Ara?**						Wang 2014[13]					
	C1	/	/	C2	s/N.A.	107.1	89.8				
	C2	/	/	C3	N.A/N.A	89.8	76.8				
	C3	77.8	78	C4	N.A/N.A	76.8	78.6				
	C4	/	/	C5	s/N.A.	78.6	70.7				
**Unk9 - Glc?**						Laguri 2018[11]					
	C1	90.1	74.3	C2	s/N.A.	103.3	74.3				
	C2	74.3	70.1	C3		74.3	70.8				
**Unk10 - Glc?**						Kang 2018[14]					

	C1	/	/	C2	s/N.A.	101.2	70.1				
	C2	/	/	C3	N.A/N.A	70.1	84.5				
	C3	/	/	C4	N.A/N.A	84.5	67.7				
	C4	67.2	71.2	C5	N.A/N.A	67.7	71.5				
	C5	71.3	60.1	C6	s/N.A.	71.5	60.5				
**Unk11 - Rha?**						*Tan 2010*[18]					
	C1	98.6	68.8	C2	s/N.A.	100.7	70.5				
	C2	68.8	70.2	C3	N.A/N.A	70.5	70.6				
**Unk12**											
	C1	97.1	73.2	C2	s/N.A.						
	C2	73.2	67.6	C3	N.A/N.A						
	C3	67.6	70.1	C4	N.A/N.A						
	C4	70.1	64	C5	s/N.A.						
**Unk13**											
	C1	105.3	63.6	C2	N.A/N.A						
	C2	63.6	72.6	C3	N.A/N.A						
**Unk14**											
		104.4	62.1		s/N.A.						
**Unk15 - Gal?**						*Phyo 2017a*[22]					
		98.6	67.7		s/N.A.	98.4	68.8				
**Unk16 - Ara?**						*Gorin 1975*[12]		*Rondeau-M. 2008*[8]			
		106.3	73.5		s/N.A.	105.1	71.8	105.5	72.9		
**Unk17 - Xyl?**						*Hollman 2009*[26]		*Gorin 1975*[12]			
		68.7	66.2		N.A/N.A	69.3	65.4	69.4	67.3		
**Unk18 - Glc?**						*Pfeffer 1984*[10]					
		71	64.4		N.A/N.A	71.6	63.7				
**Unk19 - Glc?**						*Kang 2018*[14]					
		69.5	61.3		N.A/N.A	71.7	60.5				
**Unk20 - Man?**						*Marchessault 1990*[27]		*Gorin 1975*[12]		*Hantus 1997*[25]	
		71.4	63.5		N.A/N.A	70.9	62.9	71.7	62.5	72.1	62.7
**Unk21**											
		99.9	71.9		s/N.A.						

Table S7. **Details of the different methods used for quantification. Method 1** is the averaged carbon intensity for one type of glycan (see Equation 1). It considers the carbon numbers integrated. For each spin system, we considered the non-overlapping and the same type of glycan overlapping peaks, and averaged them, then we summed these averages according to the glycan type. **Method 2** uses **method 1**, normalizing the integrals by the number of spin systems assigned for a particular glycan (see Equation 2). Integrals are compared with quantitative mass spectrometry, **method 2** being the one considering the spin system number and, therefore, being the most accurate.

		**Method 1**								**Method 2**				**MS**
		Σ(int)	**Σ(int) COR.[a]**	nC no overlap	nC overlap	tot. nC	int/nC	%int/nC	**%int/nC COR.[a]**	**x** spin system	(int/ncarbon)***x**	%norm	**%norm COR.[a]**	
**Starch**	overall	1.1E+09	3.7E+09	42	10	52	7.1E+07	**30.7**	**60.8**	7	4.9E+08	**43.6**	**73.0**	**85**
	polysaccharide	8.5E+08	3.0E+09	28	4	32	9.3E+07	21.6	43.4	4	3.7E+08	33.4	56.6	
	monosac./end-group	2.0E+08	6.9E+08	14	6	20	3.5E+07	8.0	16.1	3	1.0E+08	9.3	15.7	
**Glc**	overall	1.7E+08	1.7E+08	14	0	14	1.2E+07	**18.4**	**10.4**	4	4.8E+07	**14.9**	**7.1**	
	polysaccharide	1.1E+08	1.1E+08	10	0	10	1.1E+07	8.7	5.0	3	3.2E+07	10.1	4.9	
	monosac./end-group	6.2E+07	6.2E+07	4	0	4	1.6E+07	12.6	7.3	1	1.6E+07	4.9	2.4	
**Gal**	overall	2.3E+08	2.3E+08	12	0	12	1.9E+07	**29.3**	**16.6**	3	5.8E+07	**17.8**	**8.5**	
	polysaccharide	1.4E+08	1.4E+08	8	0	8	1.7E+07	14.1	8.1	2	3.5E+07	10.9	5.3	**8.2**
	monosac./end-group	9.2E+07	9.2E+07	4	0	4	2.3E+07	18.6	10.7	1	2.3E+07	7.2	3.5	
**Rha**	overall	1.3E+08	1.3E+08	28	0	28	4.8E+06	**7.3**	**4.1**	8	3.8E+07	**11.8**	**5.6**	
	polysaccharide	1.3E+08	1.3E+08	28	0	28	4.8E+06	3.9	2.2	8	3.8E+07	12.0	5.8	**3.7**
	monosac./end-group	0.0E+00	0.0E+00	0	0	0	0.0E+00	0.0	0.0	0	0.0E+00	0.0	0.0	
**Man**	overall	1.3E+08	1.3E+08	20	8	28	4.5E+06	**6.8**	**3.9**	3	1.3E+07	**4.2**	**2.0**	
	polysaccharide	1.0E+08	1.0E+08	16	2	18	5.6E+06	4.6	2.6	2	1.1E+07	3.5	1.7	**1.4**
	monosac./end-group	2.5E+07	2.5E+07	4	6	10	2.5E+06	2.0	1.1	1	2.5E+06	0.8	0.4	
**Xyl**	overall	1.0E+08	1.0E+08	28	10	38	2.7E+06	**4.1**	**2.3**	6	1.6E+07	**5.0**	**1.4**	
	polysaccharide	1.0E+08	1.0E+08	28	10	38	2.7E+06	2.2	1.2	6	1.6E+07	5.0	1.4	**1.1**
	monosac./end-group	0.0E+00	0.0E+00	0	0	0	0.0E+00	0.0	0.0	0	0.0E+00	0.0	0.0	
**Ara**	overall	5.9E+07	5.9E+07	16	10	26	2.3E+06	**3.5**	**2.0**	4	9.1E+06	**2.8**	**1.3**	
	polysaccharide	5.0E+07	5.0E+07	12	10	22	2.3E+06	1.8	1.1	3	6.8E+06	2.1	1.0	**0.8**
	monosac./end-group	9.3E+06	9.3E+06	4	0	4	2.3E+06	1.9	1.1	1	2.3E+06	0.7	0.4	

correcting factor[a] **3.50**

[a] A correction factor of 3.50 (obtained from the 1D spectra presented in **Figure S8**) was applied to the starch intensities.

Table S8. **^{13}C raw integrals used for quantification of *P. beijerinckii* polysaccharides.** As mentioned in the main text, integrals are color-coded in red and orange according to the type of overlap: overlaps between different types of glycans are in red while overlaps among the same kind of glycan are in orange. "p" and "m" are used to differentiate polysaccharide and monosaccharide/end-groups, respectively. To differentiate those two types of glycan units, we used the carbon 1 chemical shift and CCMRD database.

	peak int.		SQ	SQ		peak int.	av. unit	mono./poly.
**starch a**	1.8E+07	C1	101.3	73.9	C2	2.0E+07	1.39E+07	***p***
	1.3E+07	C2	73.9	75.4	C3	1.6E+06		
	**3.7E+05**	C3	75.4	76.6	C4	**2.8E+06**		
	1.6E+07	C4	76.6	70.5	C5	1.4E+07		
	1.6E+07	C5	70.5	62.7	C6	1.3E+07		
**starch b**	**1.1E+08**	C1	100.2	72.4	C2	**9.2E+07**	3.96E+07	***p***
	**7.3E+07**	C2	72.4	74	C3	**7.4E+07**		
	**4.3E+07**	C3	74	77.7	C4	**3.5E+07**		
	4.0E+07	C4	77.7	71.8	C5	3.9E+07		
	**4.7E+07**	C5	71.8	61.3	C6	**7.2E+07**		
**starch c**	2.3E+07	C1	99	72	C2	1.1E+07	1.48E+07	***p***
	**7.3E+06**	C2	72	76	C3	**1.6E+07**		
	1.3E+07	C3	76	74.7	C4	1.8E+07		
	1.2E+07	C4	74.7	70.9	C5	1.2E+07		
	1.1E+07	C5	70.9	62	C6	1.8E+07		
**starch-as-Glc1**	4.6E+07	C1	96.6	75	C2	2.9E+07	3.75E+07	***p***
	**3.7E+05**	C2	75	76.6	C3	**2.8E+06**		
	**2.0E+07**	C3	76.6	70.4	C4	**1.7E+07**		
	**1.6E+07**	C4	70.4	72	C5	**1.6E+07**		
	**4.7E+07**	C5	72	61.4	C6	**7.2E+07**		
**starch-as-Glc2**	**3.1E+07**	C1	92.7	72.4	C2	**2.0E+07**	7.23E+06	***m***
	7.3E+06	C2	72.4	75.4	C3	1.6E+07		
	5.0E+06	C3	75.4	77.6	C4	8.3E+05		
	**5.1E+06**	C4	77.6	76.8	C5	**7.3E+06**		
	**2.1E+07**	C5	76.8	61.5	C6	**3.0E+07**		
**starch-end1**	**1.1E+08**	C1	100.3	72.3	C2	**9.2E+07**	2.05E+07	***m***
	**7.3E+07**	C2	72.3	73.7	C3	**7.4E+07**		
	**3.7E+07**	C3	73.7	70.1	C4	**2.7E+07**		
	1.6E+07	C4	70.1	73.5	C5	1.6E+07		
	1.2E+07	C5	73.5	61.3	C6	3.8E+07		
**starch-end2**	**1.1E+08**	C1	100.2	72.4	C2	**9.2E+07**	1.45E+07	***m***
	**4.2E+07**	C2	72.4	74	C3	**3.3E+07**		
	2.1E+07	C3	74	77.7	C4	1.7E+07		
	6.4E+06	C4	77.9	75.1	C5	7.0E+06		
	1.5E+07	C5	75.1	61.4	C6	2.1E+07		
**Glucan 1**		C1	/	/	C2		2.21E+07	***p***
		C2	/	/	C3			
		C3	/	/	C4			
	7.2E+06	C4	69.4	75.7	C5	8.1E+06		
	2.9E+07	C5	75.7	61.7	C6	4.4E+07		
**Glucan 2**	**6.0E+06**	C1	104	70.1	C2	**8.9E+06**	3.84E+06	***p***
	3.8E+06	C2	70.1	82.4	C3	3.9E+06		
	**2.8E+06**	C3	82.4	64.8	C4	**3.5E+06**		
	**6.6E+06**	C4	64.8	74.1	C5	**3.5E+06**		
	**6.6E+06**	C5	74.1	64.8	C6	**3.5E+06**		
**Glucan3**	5.2E+05	C1	102.7	73.7	C2	2.1E+06	2.81E+06	***p***

	4.3E+06	C2	73.7	67.1	C3	4.3E+06		
**Glucose1**	6.0E+06	C1	94.6	71.7	C2	4.7E+06	1.56E+07	***m***
	2.4E+07	C2	71.7	73.4	C3	2.7E+07		
	**2.7E+07**	C3	73.4	69.4	C4	**4.2E+07**		
	**2.7E+07**	C4	69.4	73.4	C5	**4.2E+07**		
	**1.8E+07**	C5	73.4	61.4	C6	**3.8E+07**		
**Ara1**	6.4E+06	C1	107.9	81.7	C2	3.8E+06	5.10E+06	***p***
	**2.4E+06**	C2	81.7	77.8	C3	**2.9E+06**		
	**2.5E+06**	C3	77.8	82.2	C4	**1.6E+06**		
	**3.4E+06**	C4	82.2	64.7	C5	**3.4E+06**		
**Ara2**	**6.4E+06**	C1	108	81.7	C2	**3.8E+06**	0	***p***
	**4.1E+06**	C2	81.7	75.2	C3	**4.3E+06**		
	**4.1E+06**	C3	75.2	81.3	C4	**4.3E+06**		
	**8.6E+06**	C4	81.3	63.1	C5	**1.4E+07**		
**Ara3**		C1	/	/	C2		7.47E+06	***p***
	8.7E+06	C2	89	74.8	C3	6.3E+06		
	**4.1E+06**	C3	74.8	81.6	C4	**4.3E+06**		
	**8.6E+06**	C4	81.6	63	C5	**1.4E+07**		
**Ara4**		C1	/	/	C2		2.33E+06	***m***
	**5.1E+06**	C2	78	77	C3	**7.3E+06**		
	2.3E+06	C3	77	83	C4	2.7E+06		
	2.1E+06	C4	83	71	C5	2.1E+06		
**Gal1**	**6.0E+06**	C1	104	70.3	C2	**8.9E+06**	2.52E+07	***p***
	**9.0E+06**	C2	70.3	72.2	C3	**8.3E+06**		
	3.4E+07	C3	72.2	76.3	C4	4.7E+07		
	**2.0E+07**	C4	76.3	70.5	C5	**1.7E+07**		
	8.7E+06	C5	70.5	61.3	C6	1.1E+07		
**Gal2**	2.24E+07	C1	98.4	72.2	C2	2.08E+07	2.30E+07	***m***
	**4.2E+07**	C2	72.2	73.9	C3	**3.2E+07**		
	2.2E+07	C3	73.9	77.9	C4	1.9E+07		
	**2.5E+06**	C4	77.9	76.7	C5	**3.1E+06**		
	2.1E+07	C5	76.7	61.6	C6	3.0E+07		
**Gal3**	**6.0E+06**	C1	104	70.2	C2	**8.9E+06**	9.63E+06	***p***
	4.0E+06	C2	70.3	82.4	C3	3.4E+06		
	**2.8E+06**	C3	82.4	65.1	C4	**3.5E+06**		
	**6.6E+06**	C4	65.1	74	C5	**3.5E+06**		
	1.7E+07	C5	74	70	C6	1.4E+07		
**a-Man1**	9.8E+06	C1	99.7	69.1	C2	7.4E+06	8.63E+06	***p***
	**5.4E+06**	C2	69.1	72.8	C3	**5.2E+06**		
	**9.1E+06**	C3	72.8	74.9	C4	**1.0E+07**		
	**1.0E+07**	C4	74.9	73	C5	**9.1E+06**		
	**1.3E+06**	C5	73	60.9	C6	**5.2E+06**		
**a-Man2**	2.8E+06	C1	93.1	69.1	C2	2.1E+06	2.47E+06	***m***
	**5.4E+06**	C2	69.1	72.8	C3	**5.2E+06**		
	**9.1E+06**	C3	72.8	74.9	C4	**1.0E+07**		
	**1.0E+07**	C4	74.9	73	C5	**9.1E+06**		
	**1.3E+06**	C5	73	60.9	C6	**5.2E+06**		
**b-Man1**	7.0E+06	C1	102.1	63.7	C2	1.0E+07	5.91E+06	***p***
	3.7E+06	C2	63.7	73.4	C3	1.8E+06		
	**2.7E+07**	C3	73.4	69.5	C4	**4.2E+07**		
	1.4E+07	C4	69.5	74.1	C5	8.7E+06		
	8.2E+05	C5	74.1	66.1	C6	5.0E+05		
**Rha1**	3.3E+06	C1	99.6	80.3	C2	3.3E+06	2.64E+06	***p***

	1.5E+06	C2	80.3	72	C3	2.6E+06		
	**7.5E+06**	C3	72	70.4	C4	**6.8E+06**		
	3.8E+06	C4	70.4	68.2	C5	5.0E+06		
	5.5E+05	C5	68.2	20.5	C6	1.1E+06		
**Rha2**	1.6E+06	C1	101.1	75.8	C2	2.6E+06	4.55E+06	***p***
	1.6E+06	C2	75.8	73.2	C3	5.4E+05		
	**2.0E+07**	C3	73.2	71.6	C4	**1.8E+07**		
	3.9E+06	C4	71.6	69.4	C5	4.8E+06		
	4.7E+06	C5	69.4	20.9	C6	1.7E+07		
**Rha3**	2.9E+06	C5	69	20.8	C6	1.7E+07	9.76E+06	***p***
**Rha4**	9.5E+06	C5	69.9	17.3	C6	1.9E+07	1.42E+07	***p***
**Rha5**	4.9E+06	C5	67.7	19.5	C6	6.2E+06	5.51E+06	***p***
**Rha6**	1.2E+06	C5	66.6	20.2	C6	2.6E+06	1.92E+06	***p***
**Rha7**	2.2E+06	C5	67.6	17.3	C6	3.6E+06	2.90E+06	***p***
**Rha8**	1.5E+06	C5	67.3	16.8	C6	6.3E+06	3.91E+06	***p***
**Xyl1**	**1.7E+06**	C1	103.9	71.5	C2	**1.3E+06**	0	***p***
	**7.1E+06**	C2	71.5	70.1	C3	**4.9E+06**		
	**2.7E+07**	C3	70.1	73.4	C4	**3.7E+07**		
	**1.8E+07**	C4	73.4	61.3	C5	**3.8E+07**		
**Xyl2**	**1.7E+06**	C1	104	71.6	C2	**1.3E+06**	3.77E+06	***p***
	**4.4E+06**	C2	71.6	73.2	C3	**7.2E+06**		
	4.8E+06	C3	73.2	67.6	C4	1.3E+06		
	4.0E+06	C4	67.6	63.6	C5	5.0E+06		
**Xyl3**	**1.7E+06**	C1	103.9	71.5	C2	**1.3E+06**	6.96E+06	***p***
	**2.0E+07**	C2	71.5	73.4	C3	**1.8E+07**		
	**2.7E+07**	C3	73.4	69.3	C4	**4.2E+07**		
	8.1E+06	C4	69.3	62.4	C5	5.8E+06		
**Xyl-2fold4**	**1.7E+06**	C1	103.9	71.5	C2	**1.3E+06**	4.65E+06	***p***
	**4.4E+06**	C2	71.5	73.5	C3	**7.2E+06**		
	2.7E+06	C3	73.5	84.9	C4	1.3E+06		
	6.0E+06	C4	84.9	61.6	C5	8.6E+06		
**Xyl-2fold5**	2.4E+06	C1	102.5	76.2	C2	2.7E+06	3.16E+06	***p***
	7.1E+06	C2	76.2	75.3	C3	2.4E+06		
	2.7E+06	C3	75.3	81.3	C4	2.9E+06		
	3.9E+06	C4	81.3	67.5	C5	1.1E+06		
**Xyl-2fold6**	2.9E+06	C1	104.8	72	C2	4.8E+06	2.31E+06	***p***
	**7.3E+06**	C2	72	75.8	C3	**1.6E+07**		
	1.3E+06	C3	75.8	82	C4	1.1E+06		
	7.9E+05	C4	82	61.8	C5	2.9E+06		
**Ac-Met1**	9.0E+06	Met1	174.4	21.2	Ac1	1.2E+07	1.06E+07	***p***
**Ac-Met2**	9.5E+06	Met2	173.8	20.8	Ac2	2.0E+07	1.48E+07	***p***
**Unk1 - Gal?**	1.2E+07	C1	98.7	64.9	C2	2.2E+07	9.90E+06	***m***
	**6.6E+06**	C2	64.9	74	C3	**3.5E+06**		
	**2.2E+07**	C3	74	77.9	C4	**1.9E+07**		
	3.5E+06	C4	77.9	70.7	C5	8.2E+06		
	2.3E+06	C5	70.7	61.3	C6	1.1E+07		
**Unk2 - Gal?**	4.4E+06	C1	95.1	72.3	C2	2.5E+06	3.00E+06	***m***
	**7.3E+06**	C2	72.3	75.6	C3	**1.6E+07**		
	5.0E+06	C3	75.6	77.5	C4	8.3E+05		
	**2.5E+06**	C4	77.5	77.3	C5	**4.2E+06**		
	3.8E+06	C5	77.3	68.3	C6	1.3E+06		
**Unk3 - Gal?**	6.3E+06	C1	88.4	74.1	C2	4.0E+06	1.99E+07	***m***
	1.7E+07	C2	74.1	69.5	C3	1.4E+07		

	7.2E+06	C3	69.5	75.6	C4	8.1E+06		
	1.6E+07	C4	75.6	72.1	C5	7.3E+06		
	4.7E+07	C5	72.1	61.3	C6	7.2E+07		
**Unk4 - Ara?**	6.4E+06	C3	71	86.05	C4	4.2E+06	8.42E+06	***m***
	9.7E+06	C4	86.05	62.06	C5	1.3E+07		
**Unk5 - Met-Ara?**	4.3E+06	C1	102.2	71	C2	2.2E+06	2.26E+07	***p***
	1.1E+07	C2	71.2	67.2	C3	3.6E+07		
	3.6E+07	C3	67	71.6	C4	1.6E+07		
	3.3E+07	C4	71.3	63	C5	4.3E+07		
**Unk6 - Ara?**		C1	/	/	C2		2.24E+06	***m***
	2.7E+06	C2	82	76.8	C3	2.3E+06		
	2.3E+06	C3	76.8	82	C4	2.7E+06		
	1.5E+06	C4	82	63.2	C5	1.8E+06		
**Unk7 - Ara?**		C1	/	/	C2		2.42E+06	***m***
	4.7E+06	C2	62.1	73.6	C3	2.6E+06		
	2.7E+06	C3	73.6	83.7	C4	1.3E+06		
	1.5E+06	C4	83.7	61.5	C5	1.7E+06		
**Unk8 - Ara?**		C1	/	/	C2		9.21E+05	***m***
		C2	/	/	C3			
	9.2E+05	C3	77.8	78	C4	9.2E+05		
		C4	/	/	C5			
**Unk9 - Glc?**	8.0E+06	C1	90.1	74.3	C2	5.4E+06	2.49E+07	***m***
	2.4E+07	C2	74.3	70.1	C3	6.2E+07		
**Unk10 - Glc?**		C1	/	/	C2		1.25E+07	***m***
		C2	/	/	C3			
		C3	/	/	C4			
	3.6E+07	C4	67.2	71.2	C5	1.1E+07		
	1.1E+06	C5	71.3	60.1	C6	2.3E+06		
**Unk11 - Rha?**	1.2E+07	C1	98.6	68.8	C2	1.2E+07	7.21E+06	***m***
	3.8E+06	C2	68.8	70.2	C3	8.9E+05		
**Unk12**	6.6E+06	C1	97.1	73.2	C2	2.5E+06	4.84E+06	***m***
	4.8E+06	C2	73.2	67.6	C3	2.3E+02		
	9.5E+05	C3	67.6	70.1	C4	1.7E+06		
	8.3E+06	C4	70.1	64	C5	1.4E+07		
**Unk13**	1.9E+06	C1	105.3	63.6	C2	2.9E+06	2.30E+07	***p***
	5.2E+07	C2	63.6	72.6	C3	3.6E+07		
**Unk14**	2.6E+06	Unk14	104.4	62.1	Unk14	4.1E+06	3.34E+06	***p***
**Unk15 - Gal?**	2.3E+06	Unk15	98.6	67.7	Unk15	3.1E+06	2.68E+06	***p***
**Unk16 - Ara?**	1.9E+06	Unk16	106.3	73.5	Unk16	3.1E+06	2.51E+06	***p***
**Unk17 - Xyl?**	7.1E+05	Unk17	68.7	66.2	Unk17	1.7E+06	1.21E+06	***m***
**Unk18 - Glc?**	2.7E+02	Unk18	71	64.4	Unk18	1.5E+06	7.74E+05	***m***
**Unk19 - Glc?**	2.1E+06	Unk19	69.5	61.3	Unk19	1.2E+06	1.67E+06	***m***
**Unk20 - Man?**	1.4E+07	Unk20	71.4	63.5	Unk20	9.8E+06	1.17E+07	***m***
**Unk21**	1.2E+07	Unk21	99.9	71.9	Unk21	2.7E+06	7.16E+06	***p***

Table S9. **List of correlations found in the different experiments.** CP-INADEQUATE experiments allow good chemical shift dispersion and detect only rigid molecules. Peaks detected in the SQ experiments: CORD, which identify short-range correlations in molecules with long T_2s; PAR, which correspond to intramolecular through space correlations; RFDR, which detects intra- and intermolecular trough-space correlations and (PAR-0.4*CORD)) which single-out intermolecular through space contacts.

Glycan residue	SQ	SQ	Contact	CP-INAD	RFDR 1.5ms	CORD 53ms	PAR 14ms	PAR-0.4*CORD
**starch a**	101.3	73.9	C1/C2	x	x	x	x	
	75.4	73.9	C3/C2	x		x	x	
	76.6	75.4	C4/C3	x	x	x	x	
	76.6	70.5	C4/C5	x	x	x	x	
	70.5	62.7	C5/C6	x	x	x		
**starch b**	100.2	72.4	C1/C2	x	x	x	x	
	74.0	72.4	C3/C2		x	x	x	
	77.7	74.0	C4/C3		x	x	x	
	77.7	71.8	C4/C5		x	x	x	
	71.8	61.3	C5/C6	x	x	x	x	
**starch c**	99.0	72.0	C1/C2	x	x	x	x	
	76.0	72.0	C3/C2	x	x	x	x	
	76.0	74.7	C3/C4	x			x	
	74.7	70.9	C4/C5	x	x	x	x	
	70.9	62.0	C5/C6	x	x	x	x	
**starch-as-Glc1**	96.6	75.0	C1/C2		x			
	76.6	75.0	C3/C2	x			x	
	76.6	70.4	C3/C4	x	x	x	x	
	72.0	70.4	C5/C4	x		x	x	
	72.0	61.4	C5/C6	x	x	x	x	
**starch-as-Glc2**	92.7	72.4	C1/C2					
	75.4	72.4	C3/C2	x	x	x	x	
	77.6	75.4	C4/C3	x				
	77.6	76.8	C4/C5					
	76.8	61.5	C5/C6		x		x	
**starch-end1**	100.3	72.3	C1/C2	x	x	x	x	
	73.7	72.3	C3/C2	x		x	x	
	73.7	70.1	C3/C4	x		x	x	
	73.5	70.1	C5/C4	x	x	x	x	
	73.5	61.3	C5/C6	x	x	x	x	
**starch-end2**	100.2	72.4	C1/C2	x		x	x	
	74.0	72.4	C3/C2	x	x	x	x	
	77.7	74.0	C4/C3		x	x	x	
	77.9	75.1	C4/C5	x				
	75.1	61.4	C5/C6			x	x	x
**Glucan 1**	75.7	69.4	C5/C4					
	75.7	61.7	C5/C6			x		
**Glucan 2**	104.0	70.1	C1/C2					
	82.4	70.1	C3/C2					
	82.4	64.8	C3/C4					
	74.1	64.8	C5/C4					
	74.1	64.8	C5/C6					
**Glucan3**	102.7	73.7	C1/C2					
	73.7	67.1	C3/C2					

**Glucose1**	94.6	71.7	C1/C2			
	73.4	71.7	C3/C2		x	x
	73.4	69.4	C3/C4			x
	73.4	69.4	C5/C4			x
	73.4	61.4	C5/C6	x	x	x
**Ara1**	107.9	81.7	C1/C2			
	81.7	77.8	C2/C3			
	82.2	77.8	C4/C3			
	82.2	64.7	C4/C5			
**Ara2**	108.0	81.7	C1/C2			
	81.7	75.2	C2/C3			
	81.3	75.2	C4/C3			
	81.3	63.1	C4/C5			
**Ara3**	89	74.8	C2/C3			
	81.6	74.8	C4/C3			
	81.6	63	C4/C5			
**Ara4**	78	77	C2/C3			
	83	77	C4/C3			
	83	71	C4/C5			
**Gal1**	104	70.3	C1/C2			
	72.2	70.3	C3/C2	x		x
	76.3	72.2	C4/C3	x	x	x
	76.3	70.5	C4/C5	x	x	x
	70.5	61.3	C5/C6	x		x
**Gal2**	98.4	72.2	C1/C2			
	73.9	72.2	C3/C2			
	77.9	73.9	C4/C3			
	77.9	76.7	C4/C5			
	76.7	61.6	C5/C6			
**Gal3**	104	70.2	C1/C2	x		x
	82.4	70.3	C3/C2		x	x
	82.4	65.1	C3/C4			
	74	65.1	C5/C4		x	
	74	70	C5/C6			
**a-Man1**	99.7	69.1	C1/C2			
	72.8	69.1	C3/C2	x		
	74.9	72.8	C4/C3	x		x
	74.9	73	C4/C5	x		x
	73	60.9	C5/C6	x		x
**a-Man2**	93.1	69.1	C1/C2			
	72.8	69.1	C3/C2	x		
	74.9	72.8	C4/C3	x		x
	74.9	73	C4/C5	x		x
	73	60.9	C5/C6	x		x
**b-Man1**	102.1	63.7	C1/C2			
	73.4	63.7	C3/C2		x	
	73.4	69.5	C3/C4			x
	74.1	69.5	C5/C4			
	74.1	66.1	C5/C6			
**Rha1**	99.6	80.3	C1/C2			
	80.3	72	C2/C3			
	72	70.4	C3/C4	x		x
	70.4	68.2	C4/C5			

	68.2	20.5	C5/C6					
**Rha2**	101.1	75.8	C1/C2			x		
	75.8	73.2	C2/C3	x				
	73.2	71.6	C3/C4			x		
	71.6	69.4	C4/C5	x				
	69.4	20.9	C5/C6					
**Rha3**	69	20.8	C5/C6					
**Rha4**	69.9	17.3	C5/C6					
**Rha5**	67.7	19.5	C5/C6					
**Rha6**	66.6	20.2	C5/C6					
**Rha7**	67.6	17.3	C5/C6					
**Rha8**	67.3	16.8	C5/C6					
**Xyl1**	103.9	71.5	C1/C2					
	71.5	70.1	C2/C3	x		x		
	73.4	70.1	C4/C3					
	73.4	61.3	C4/C5					
**Xyl2**	104	71.6	C1/C2					
	73.2	71.6	C3/C2			x		
	73.2	67.6	C3/C4					
	67.6	63.6	C4/C5					
**Xyl3**	103.9	71.5	C1/C2					
	73.4	71.5	C3/C2			x		
	73.4	69.3	C3/C4			x		
	69.3	62.4	C4/C5					
**Xyl-2fold4**	103.9	71.5	C1/C2	x				
	73.5	71.5	C3/C2			x		
	84.9	73.5	C4/C3	x				
	84.9	61.6	C4/C5	x				
**xyl-2fold5**	102.5	76.2	C1/C2	x				
	76.2	75.3	C2/C3	x				
	81.3	75.3	C4/C3	x	x	x		
	81.3	67.5	C4/C5	x		x		
**Xyl-2fold6**	104.8	72	C1/C2					
	75.8	72	C3/C2	x		x		
	82	75.8	C4/C3					
	82	61.8	C4/C5					x
**Intra. J>1**								
**Starch a**	101.3	75.4	C1a/3a			x	x	x
	101.3	76.6	C1a/4a			x	x	
	101.3	70.5	C1a/5a			x	x	
	101.3	62.7	C1a/6a			x	x	
	76.6	73.9	C4a/2a				x	
	73.9	70.5	C2a/5a			x	x	
	73.9	62.7	C2a/6a				x	
	75.4	70.5	C3a/5a			x	x	
	75.4	62.7	C3a/6a			x	x	
	76.6	62.7	C4a/6a			x	x	
**Starch b**	100.2	74.0	C1b/3b			x	x	
	100.2	77.7	C1b/4b					
	100.2	71.8	C1b/5b			x	x	x
	100.2	61.3	C1b/6b			x	x	
	77.7	72.4	C4b/2b				x	
	72.4	71.8	C2b/5b			x	x	
	72.4	61.3	C2b/6b			x	x	

	74.0	71.8	C3b/5b	x	x	
	74.0	61.3	C3b/6b	x	x	
	77.7	61.3	C4b/6b		x	
**Starch c**	99.0	76.0	C1c/3c	x	x	
	99.0	74.7	C1c/4c	x	x	
	99.0	70.9	C1c/5c	x	x	
	99.0	62.0	C1c/6c	x	x	
	74.7	72.0	C4c/2c	x	x	
	72.0	70.9	C2c/5c		x	
	72.0	62.0	C2c/6c	x	x	x
	76.0	70.9	C3c/5c	x	x	
	76.0	62.0	C3c/6c	x	x	
	74.7	62.0	C4c/6c	x	x	
**Starch-as-Glc1**	96.6	76.6	C1/3			
	96.6	70.4	C1/4			
	96.6	72.0	C1/5			
	96.6	61.4	C1/6			
	75.0	70.4	C2/4	x	x	
	75.0	72.0	C2/5	x	x	
	75.0	61.4	C2/6	x	x	
	76.6	72.0	C3/5	x	x	
	76.6	61.4	C3/6		x	
	70.4	61.4	C4/6	x	x	x
**Starch-as-Glc2**	92.7	75.4	C1/3			
	92.7	77.6	C1/4			
	92.7	76.8	C1/5			
	92.7	61.5	C1/6			
	77.6	72.4	C4/2		x	
	76.8	72.4	C5/2	x	x	
	72.4	61.5	C2/6	x	x	
	76.8	75.4	C5/3	x	x	
	75.4	61.5	C3/6	x	x	
	77.6	61.5	C4/6			
**Starch-end1**	100.3	73.7	C1/3	x	x	
	100.3	70.1	C1/4	x	x	
	100.3	73.5	C1/5	x	x	
	100.3	61.3	C1/6	x	x	
	72.3	70.1	C2/4	x	x	x
	73.5	72.3	C5/2	x	x	
	72.3	61.3	C2/6	x	x	
	73.7	73.5	C3/5		x	
	73.7	61.3	C3/6	x	x	
	70.1	61.3	C4/6	x	x	
**Starch-end2**	100.2	74.0	C1/3	x	x	
	100.2	77.7	C1/4			
	100.2	75.1	C1/5		x	
	100.2	61.4	C1/6	x	x	
	77.7	72.4	C4/2		x	
	75.1	72.4	C5/2	x	x	
	72.4	61.4	C2/6	x	x	
	75.1	74.0	C5/3		x	
	74.0	61.4	C3/6	x	x	
	77.9	61.4	C4/6		x	
**Gal1**	76.3	70.3	Gal1-C4/C2	x	x	
	70.5	70.3	Gal1-C5/C2	x	x	

	70.3	61.3	Gal1-C2/C6	x	x	
	72.2	70.5	Gal1-C3/C5	x	x	
	72.2	61.3	Gal1-C3/C6	x	x	
	76.3	61.3	Gal1-C4/C6	x	x	
**a-Man1/2**	73	72.8	a-Man1/2-C5/C3	x	x	
	72.8	60.9	a-Man1/2-C3/C6	x	x	
	74.9	60.9	a-Man1/2-C4/C6	x	x	
**Xyl-2fold4**	103.9	61.6	Xyl-2fold4-C1/C5		x	
	71.5	61.6	Xyl-2fold4-C2/C5	x	x	
	75.3	61.6	Xyl-2fold4-C3/C5	x	x	
**Xyl-2fold5**	102.5	75.3	xyl-2fold5-C1/C3	x	x	
	81.3	76.2	xyl-2fold5-C4/C2		x	
**Inter. starch**	101.3	72.4	C1a/2b	x	x	x
	101.3	99	C1a/1c	x	x	x
	101.3	62	C1a/6c	x	x	x
	101.3	70.1	C1a/Starch-end1-C4			x
	75.4	69.4	C3a/Glc1-C4		x	x
	76.6	71.8	C4a/starch-C5b			x
	75	70.5	C5a/Starch-as1-C2		x	x
	100.2	70.5	C1b/5a		x	x
	100.2	62.7	C1b/C6a		x	x
	100.2	75	C1b/Starch-as1-C2		x	x
	100.2	73.5	C1b/Starch-end1-C5			x
	74	61.4	C3b/starch-as1/6			x
	99	72.4	C1c/2b	x	x	x
	99	62.7	C1c/6a	x		x
	99	61.3	C1c/6b	x	x	x
	99	73.5	C1c/Starch-end1-C5		x	x
	75.7	62	C6c/Glc1-C5		x	x
**Gal**	104	76.3	Gal3-C1/Gal1-C4		x	x
**Xyl-2fold**	103.9	103	xyl4-C1/xyl5-C1		x	
	103.9	76.2	xyl4-C1/xyl5-C2		x	x
	103.9	75.3	xyl4-C1/xyl5-C3			x
	102.5	71.5	xyl5-C1/xyl4-C2	x	x	x
	76.2	71.5	xyl5-C2/xyl4-C2	x	x	x
	75.3	71.5	xyl5-C3/xyl4-C2	x	x	x
	81.3	71.5	xyl5-C4/xyl4-C2	x		
	76.2	75.3	xyl5-C2/xyl4-C3	x	x	x
	75.3	75.3	xyl4-C3/xyl5-C3	x	x	
	81.3	75.3	xyl5-C4/xyl4-C3		x	x
	102.5	61.6	xyl5-C1/xyl4-C5	x		x
	76.2	61.6	xyl5-C2/xyl4-C5	x	x	x
	75.3	61.6	xyl5-C3/xyl4-C5	x	x	x
	81.3	61.6	xyl5-C4/xyl4-C5		x	
	67.5	61.6	xyl5-C5/xyl4-C5		x	x

References for glycan assignment

1. Arnold, A. A.; Genard, B.; Zito, F.; Tremblay, R.; Warschawski, D. E.; Marcotte, I., Identification of lipid and saccharide constituents of whole microalgal cells by ^{13}C solid-state NMR. *Biochim. Biophys. Acta Biomembr.* **2015,** *1848* (1, Part B), 369-377.
2. Arnold, A. A.; Bourgouin, J.-P.; Genard, B.; Warschawski, D. E.; Tremblay, R.; Marcotte, I., Whole cell solid-state NMR study of *Chlamydomonas reinhardtii* microalgae. *J. Biomol. NMR* **2018,** *70* (2), 123-131.
3. Poulhazan, A.; Arnold, A. A.; Warschawski, D. E.; Marcotte, I., Unambiguous Ex Situ and in Cell 2D (13)C Solid-State NMR Characterization of Starch and Its Constituents. *Int J Mol Sci* **2018,** *19* (12).
4. Rondeau-Mouro, C.; Veronese, G.; Buleon, A., High-Resolution Solid-State NMR of B-Type Amylose. *Biomacromolecules* **2006,** *7* (8), 2455-2460.
5. Paris, M.; Bizot, H.; Emery, J.; Buzaré, J. Y.; Buléon, A., Crystallinity and structuring role of water in native and recrystallized starches by ^{13}C CP-MAS NMR spectroscopy: 1: Spectral decomposition. *Carbohydrate Polymers* **1999,** *39* (4), 327-339.
6. Bradbury, J. H.; Jenkins, G. A., Determination of the structures of trisaccharides by ^{13}C NMR spectroscopy. *Carbohydr Res* **1984,** *126* (1), 125-56.
7. Roubroeks, J. P.; Andersson, R.; Mastromauro, D. I.; Christensen, B. E.; Åman, P., Molecular weight, structure and shape of oat (1→3),(1→4)- β -d-glucan fractions obtained by enzymatic degradation with (1→4)-β-d-glucan 4-glucanohydrolase from Trichoderma reesei. *Carbohydrate Polymers* **2001,** *46* (3), 275-285.
8. Rondeau-Mouro, C.; Buléon, A.; Lahaye, M., Caractérisation par RMN des biopolymères d'origine végétale, de la molécule à l'organisation supramoléculaire. *Comptes Rendus Chimie* **2008,** *11* (4), 370-379.
9. Arnold, A. A.; Genard, B.; Zito, F.; Tremblay, R.; Warschawski, D. E.; Marcotte, I., Identification of lipid and saccharide constituents of whole microalgal cells by ^{13}C solid-state NMR. *Biochimica et Biophysica Acta (BBA) - Biomembranes* **2015,** *1848* (1, Part B), 369-377.
10. Pfeffer, P. E.; Hicks, K. B.; Frey, M. H.; Opella, S. J.; Earl, W. L., Complete Solid State ^{13}C NMR Chemical Shift Assignments for α-D-Glucose, α-D-Glucose-H2O and β-D-Glucose. *Journal of Carbohydrate Chemistry* **1984,** *3* (2), 197-217.
11. Laguri, C.; Silipo, A.; Martorana, A. M.; Schanda, P.; Marchetti, R.; Polissi, A.; Molinaro, A.; Simorre, J.-P., Solid State NMR Studies of Intact Lipopolysaccharide Endotoxin. *ACS Chemical Biology* **2018,** *13* (8), 2106-2113.
12. Gorin, P. A. J.; Mazurek, M., Further Studies on the Assignment of Signals in ^{13}C Magnetic Resonance Spectra of Aldoses and Derived Methyl Glycosides. *Canadian Journal of Chemistry* **1975,** *53* (8), 1212-1223.
13. Wang, T.; Salazar, A.; Zabotina, O. A.; Hong, M., Structure and dynamics of Brachypodium primary cell wall polysaccharides from two-dimensional (13)C solid-state nuclear magnetic resonance spectroscopy. *Biochemistry* **2014,** *53* (17), 2840-54.
14. Kang, X.; Kirui, A.; Muszyński, A.; Widanage, M. C. D.; Chen, A.; Azadi, P.; Wang, P.; Mentink-Vigier, F.; Wang, T., Molecular architecture of fungal cell walls revealed by solid-state NMR. *Nature Communications* **2018,** *9* (1), 2747.
15. Dick-Pérez, M.; Zhang, Y.; Hayes, J.; Salazar, A.; Zabotina, O. A.; Hong, M., Structure and Interactions of Plant Cell-Wall Polysaccharides by Two- and Three-Dimensional Magic-Angle-Spinning Solid-State NMR. *Biochemistry* **2011,** *50* (6), 989-1000.
16. Lundborg, M.; Widmalm, G., Structural Analysis of Glycans by NMR Chemical Shift Prediction. *Analytical Chemistry* **2011,** *83* (5), 1514-1517.

17. Habibi, Y.; Heyraud, A.; Mahrouz, M.; Vignon, M. R., Structural features of pectic polysaccharides from the skin of Opuntia ficus-indica prickly pear fruits. *Carbohydr Res* **2004,** *339* (6), 1119-27.
18. Tan, L.; Varnai, P.; Lamport, D. T.; Yuan, C.; Xu, J.; Qiu, F.; Kieliszewski, M. J., Plant O-hydroxyproline arabinogalactans are composed of repeating trigalactosyl subunits with short bifurcated side chains. *J Biol Chem* **2010,** *285* (32), 24575-83.
19. Heux, L.; Hagglund, P.; Putaux, J. L.; Chanzy, H., Structural aspects in semicrystalline samples of the mannan II family. *Biomacromolecules* **2005,** *6* (1), 324-332.
20. Leung, M. Y. K.; Liu, C.; Zhu, L. F.; Hui, Y. Z.; Yu, B.; Fung, K. P., Chemical and biological characterization of a polysaccharide biological response modifier from Aloe vera L. var. chinensis (Haw.) Berg. *Glycobiology* **2004,** *14* (6), 501-510.
21. Phyo, P.; Wang, T.; Xiao, C.; Anderson, C. T.; Hong, M., Effects of Pectin Molecular Weight Changes on the Structure, Dynamics, and Polysaccharide Interactions of Primary Cell Walls of Arabidopsis thaliana: Insights from Solid-State NMR. *Biomacromolecules* **2017,** *18* (9), 2937-2950.
22. Phyo, P.; Wang, T.; Kiemle, S. N.; O'Neill, H.; Pingali, S. V.; Hong, M.; Cosgrove, D. J., Gradients in Wall Mechanics and Polysaccharides along Growing Inflorescence Stems *Plant Physiology* **2017,** *175* (4), 1593-1607.
23. Kang, X.; Kirui, A.; Dickwella Widanage, M. C.; Mentink-Vigier, F.; Cosgrove, D. J.; Wang, T., Lignin-polysaccharide interactions in plant secondary cell walls revealed by solid-state NMR. *Nature Communications* **2019,** *10* (1), 347.
24. Vignon, M. R.; Gey, C., Isolation, 1H and ^{13}C NMR studies of (4-O-methyl-d-glucurono)-d-xylans from luffa fruit fibres, jute bast fibres and mucilage of quince tree seeds. *Carbohydrate Research* **1998,** *307* (1), 107-111.
25. Hantus, S.; Pauly, M.; Darvill, A. G.; Albersheim, P.; York, W. S., Structural characterization of novel L-galactose-containing oligosaccharide subunits of jojoba seed xyloglucans. *Carbohydr Res* **1997,** *304* (1), 11-20.
26. Hollmann, J.; Elbegzaya, N.; Pawelzik, E.; Lindhauer, M. G., Isolation and characterization of glucuronoarabinoxylans from wheat bran obtained by classical and ultrasound-assisted extraction methods. *Quality Assurance and Safety of Crops & Foods* **2009,** *1* (4), 231-239.
27. Marchessault, R. H.; Taylor, M. G.; Winter, W. T., ^{13}C CP/MAS NMR spectra of poly-β-D(1 → 4) mannose: mannan. *Canadian Journal of Chemistry* **1990,** *68* (7), 1192-1195.
